# Interpretable Machine Learning Framework for Nb─Si Based Alloy Design with Enhanced Fracture Toughness

**DOI:** 10.1002/advs.75815

**Published:** 2026-05-25

**Authors:** Dezhi Chen, Chao Xu, Jingyue Yu, Qi Wang, Hongze Fang, Shuo Yin, Turab Lookman, Ruirun Chen

**Affiliations:** ^1^ National Key Laboratory For Precision Hot Processing of Metals Harbin Institute of Technology Harbin P. R. China; ^2^ Department of Mechanical Manufacturing and Biomedical Engineering Trinity College University of Dublin Dublin Ireland; ^3^ State Key Laboratory For Mechanical Behavior of Materials State Key Laboratory of Porous Metal Materials School of Materials Science and Engineering Xi'an Jiatong University Xi'an P. R. China; ^4^ AiMaterials Research LLC Santa Fe New Mexico USA

**Keywords:** machine learning, model interpretability, Nb─Si alloys, strengthening mechanisms

## Abstract

A continuing challenge in aerospace materials is the search for alloys that have desired functional properties to operate at higher temperatures with lower densities. This improves aero‐engine efficiency and reduces CO_2_ and other harmful emissions, aligning with aviation industry targets for emission reduction. Nb─Si alloys can operate at higher temperatures than current Ni‐based superalloys and have lower densities. We develop a machine learning‐driven design framework for Nb─Si based ultra‐high temperature alloys using a three‐step feature screening strategy to break the 20 MPa·m^1/2^ fracture toughness barrier. Our model predicts fracture toughness (*K*_Q_) with an error <7%, and we use SHAP(ley) and PDP analysis to interpret the model to guide alloy design. Five alloys with Si content ranging from 3 to 15 at.% were synthesized to validate model predictions. Sample #5 (Nb_38.5_Ti_38.5_Si_3_Zr_18_V_2_) achieved a *K*_Q_ of 22.791 MPa·m^1/2^, exceeding the typical range (below 20 MPa·m^1/2^) for as‐cast Nb─Si alloys. Microstructural analysis showed that the improved performance resulted from the transformation of brittle silicide phases to ductile Nbss phase and crack‐bridging toughening. Strengthening mechanism analysis reveals that solid solution strengthening was dominant (68%–84%), with excellent strength‐toughness balance.

## Graphical abstract

An interpretable machine learning framework integrating SHAP and PDP analysis identifies critical design descriptors from 139 physicochemical features for Nb─Si alloys. The framework achieves <7% prediction error and guides the discovery of Nb_38.5_Ti_38.5_Si_3_Zr_18_V_2_ alloy with 22.791 MPa·m^1/2^ fracture toughness, breaking the 20 MPa·m^1/2^ barrier. This demonstrates how feature engineering and model interpretability accelerate materials design beyond trial‐and‐error approaches.

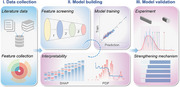

## 1 Introduction

Ultra‐high temperature alloys are critical materials for aerospace, energy, and extreme environment applications, serving as a vital part of achieving sustainable development, but still face performance optimization challenges in materials science [1, 2, 3]. More efficient aero‐engines can significantly reduce aviation emissions. Compared to nickel‐based superalloys, Nb─Si based ultra‐high temperature alloys (Nb─Si alloys) operate at approximately 200°C higher temperatures with 15% lower density, potentially improving turbine efficiency by ∼9%, reducing CO_2_ emissions by ∼9% and NO_x_ emissions by ∼6%, thus attracting considerable attention [4, 5]. However, these alloys have inherent brittleness, with room temperature fracture toughness typically below 20 MPa·m^1/2^, limiting their machinability and application in complex structural components [6]. Traditional trial‐and‐error methods no longer meet the efficiency requirements of modern materials design, whereas machine learning methods have increasingly shown potential in predicting performance and alloy optimization.

In recent years, machine learning has been successfully applied to various materials systems [7, 8, 9]. Researchers have developed active learning frameworks that combine statistical inference, adaptive design, and uncertainty assessment for materials discovery [10, 11]. This framework identifies new materials with target properties from hundreds of thousands of candidate compositions, including BaTiO_3_‐based piezoelectric ceramics and NiTi‐based shape memory alloys, performing better than traditional random search methods [12]. Zhao et al. [13]. identified V─Ti─Mo─Nb─Zr refractory high‐entropy alloys from over 3 million candidate compositions using symbolic regression‐guided optimization in 4 iterations, with a 32% improvement over previous results. Zhang et al. [14]. proposed an element substitution design strategy combining SHAP interpretability analysis and sensitivity analysis to achieve a substantial reduction of the scarce element Co in C70350 copper alloys. For Nb─Si alloys, Liu et al. [15]. optimized sample strength from 357.9 to 652.45 MPa through backpropagation artificial neural network (BP ANN) models combined with silicide design maps.

However, several challenges remain when applying machine learning to Nb─Si alloy design. First, traditional feature selection methods rely on statistical correlation without considering the physical mechanisms behind material behavior, which may select features that correlate statistically but lack physical relevance to fracture toughness. Second, machine learning models are difficult to interpret, making it hard to understand how material parameters affect predictions. Recently, methods such as SHAP (Shapley Additive exPlanations) and PDP (Partial Dependence Plots) have been utilized in materials science to interpret model predictions and guide alloy design. However, their application to Nb─Si alloy design remains limited.

To address these challenges, we employ a machine learning‐driven strategy to develop Nb─Si alloys with improved fracture toughness. Our approach includes: (1) development of a three‐step feature screening strategy that constructs 36 new features from 103 fundamental features based on materials science principles including toughness criteria, thermodynamic stability and phase formation theory, and systematically screened all 139 features; (2) comparison of machine learning models to find the best predictive model; (3) application of SHAP and PDP analysis, as well as fitting a formula to the Nb─Si alloy system in terms of how features affect performance by leveraging insights from machine learning models; (4) synthesis and characterize five validation alloys with microstructure and mechanical property characterization; (5) analysis of strengthening mechanisms and determining the contribution of each mechanism.

We synthesized five alloys with differing Si content (15, 12, 9, 6, and 3 at.%) to validate model predictions. Sample #5 (Nb_38_._5_Ti_38_._5_Si_3_Zr_18_V_2_) achieved fracture toughness of 22.791 MPa·m^1/2^ and compressive strength of 1381.37 MPa. This surpasses the traditional 20 MPa·m^1/2^ barrier, positioning our alloy as the leading as‐cast Nb─Si alloy to date.

## 2 Results and Discussion

### 2.1 Data Collection and Subset Construction

**FIGURE 1:**
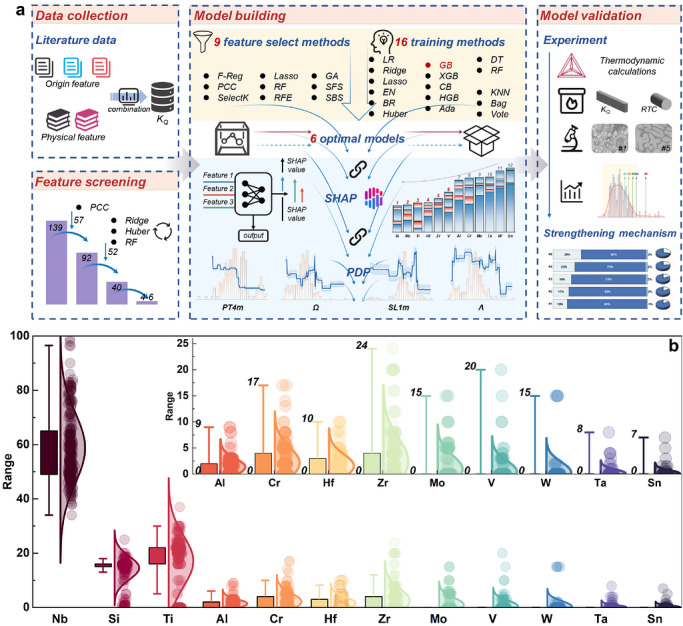
Machine learning workflow and dataset characteristics for Nb─Si alloy design. (a) The workflow includes five core modules: data collection, feature screening, model training, interpretability analysis, and model validation. (b) Element composition distribution in the Nb─Si alloy database showing frequency of occurrence for different elements.

The machine learning‐driven materials framework we use is presented in Figure 1a. The workflow includes five core modules to identify critical factors and validate predictive capability. The data collection module gathers Nb─Si alloy data from databases such as Web of Science, creating 139 feature descriptors from raw property values and domain‐informed features. The feature screening module reduces the initial features to 4–7 key features through a three‐step screening strategy. The model training module employs multiple feature selection methods combined with 16 training approaches to obtain 6 optimal predictive models. The interpretability analysis module utilizes SHAP analysis, PDP analysis, and formula fitting methods to quantify the influence of features on the target property. The model validation module synthesizes target alloys and conducts thermodynamic calculations, *K*_Q_ testing, microstructural characterization, and strengthening mechanism analysis. Figure 1b shows the elemental composition range analysis, where the Nb matrix element content ranges from 30–80 at%, Si content is distributed within 5–25 at%, and transition metal elements Ti, V, Zr, and Hf cover compositional ranges of 0–30 at%, 0–15 at%, 0–20 at%, and 0–10 at%, respectively.

### 2.2 Feature Engineering and Screening Strategy

**FIGURE 2:**
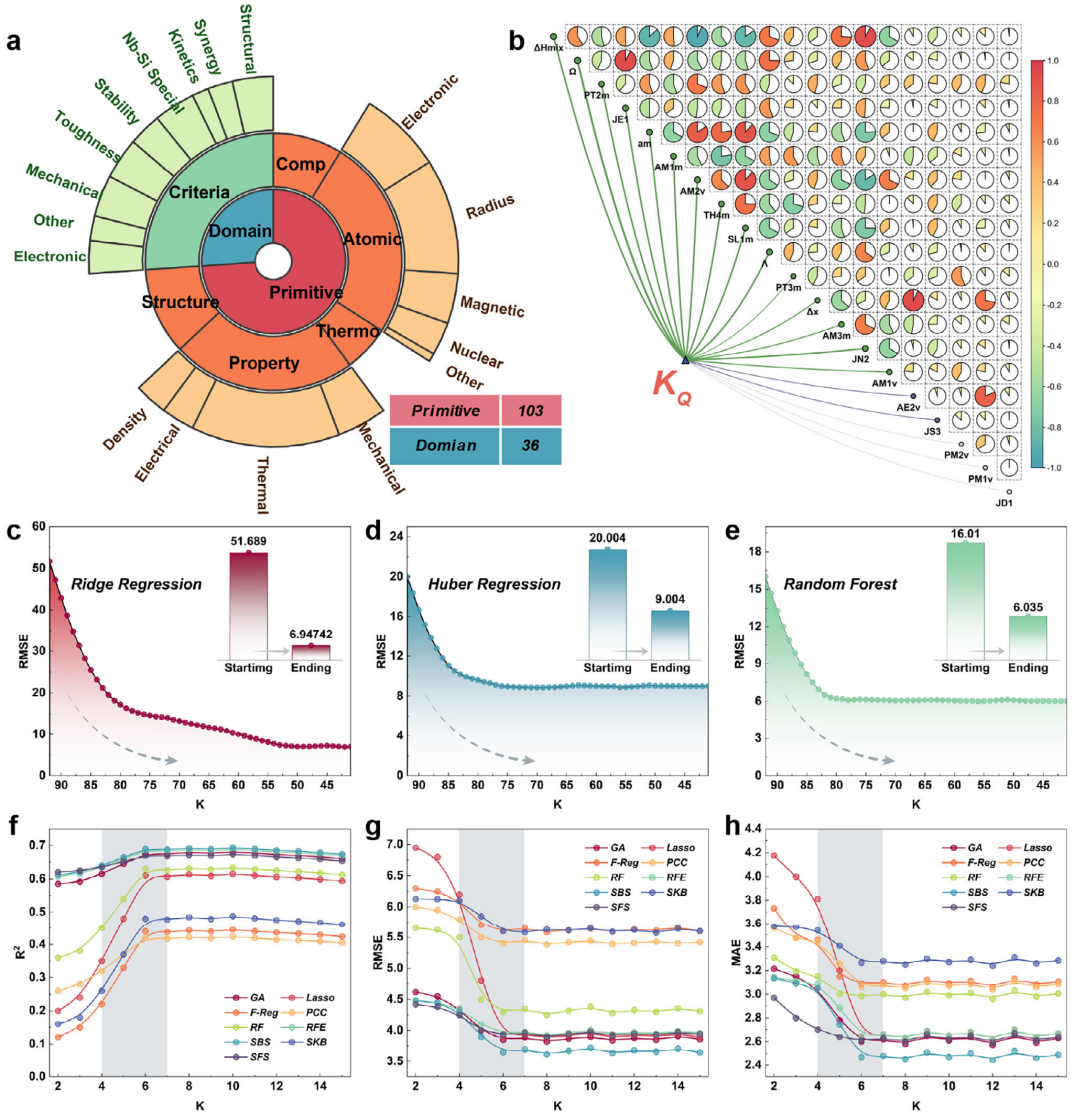
Feature screening process. (a) Feature system composition: 103 basic features and 36 domain‐informed features; (b) Top 20 key features after PCC screening; (c–e) RMSE evolution curves for three regression methods: (c) Ridge regression, (d) Huber regression, (e) Random Forest; (f–h) Performance comparison of 9 feature selection methods: (f) R^2^, (g) RMSE, (h) MAE.

A three‐step feature screening strategy was used to select key factors from 139 initial features, as shown in Figure 2. Figure 2a shows our feature system: 103 basic features and 36 domain‐informed features (Tables S1–S3). We first conducted PCC with a threshold of |r| > 0.95 to remove features with high correlations that could cause multicollinearity problems. When feature pairs exceeded this threshold, we kept the feature with stronger correlation to *K*_Q_ and removed the remainder. This process removed 48 features. Figure 2b shows the top 20 features after this screening, with colors showing their correlation strength with the target property.

Next, we used RFE with three different methods. Different regression algorithms may find different feature importance patterns, so we used Ridge regression (α = 1.0) for handling multicollinear features, Huber regression (max_iter = 200) for robustness against outliers, and Random Forest (n_estimators = 50) for capturing non‐linear feature interactions. During each elimination cycle, we evaluated the impact of removing each remaining feature through fivefold cross‐validation. For each feature, we calculated the average prediction error (mean RMSE across the three algorithms) when that feature was excluded. We removed the feature that caused the smallest increase in prediction error. This reduces to 40 features as optimal for further analysis, where model performance became stable across all three methods. Figure 2c–e shows the RMSE changes during this process, with all three methods showing similar patterns: rapid improvement as features decreased from 91 to about 40, followed by stable performance.

Finally, 9 feature selection methods were applied to the 40 features obtained from step two (Tables S4 and S5). Figure 2f–h compares performance with feature numbers varying from 2 to 15, showing R^2^ values (f), RMSE values (g), and MAE values (h). Performance saturates when feature numbers reach 4–7, indicating the likelihood of overfitting beyond this range.

### 2.3 Model Prediction Performance and SHAP‐Based Element Ranking

**FIGURE 3:**
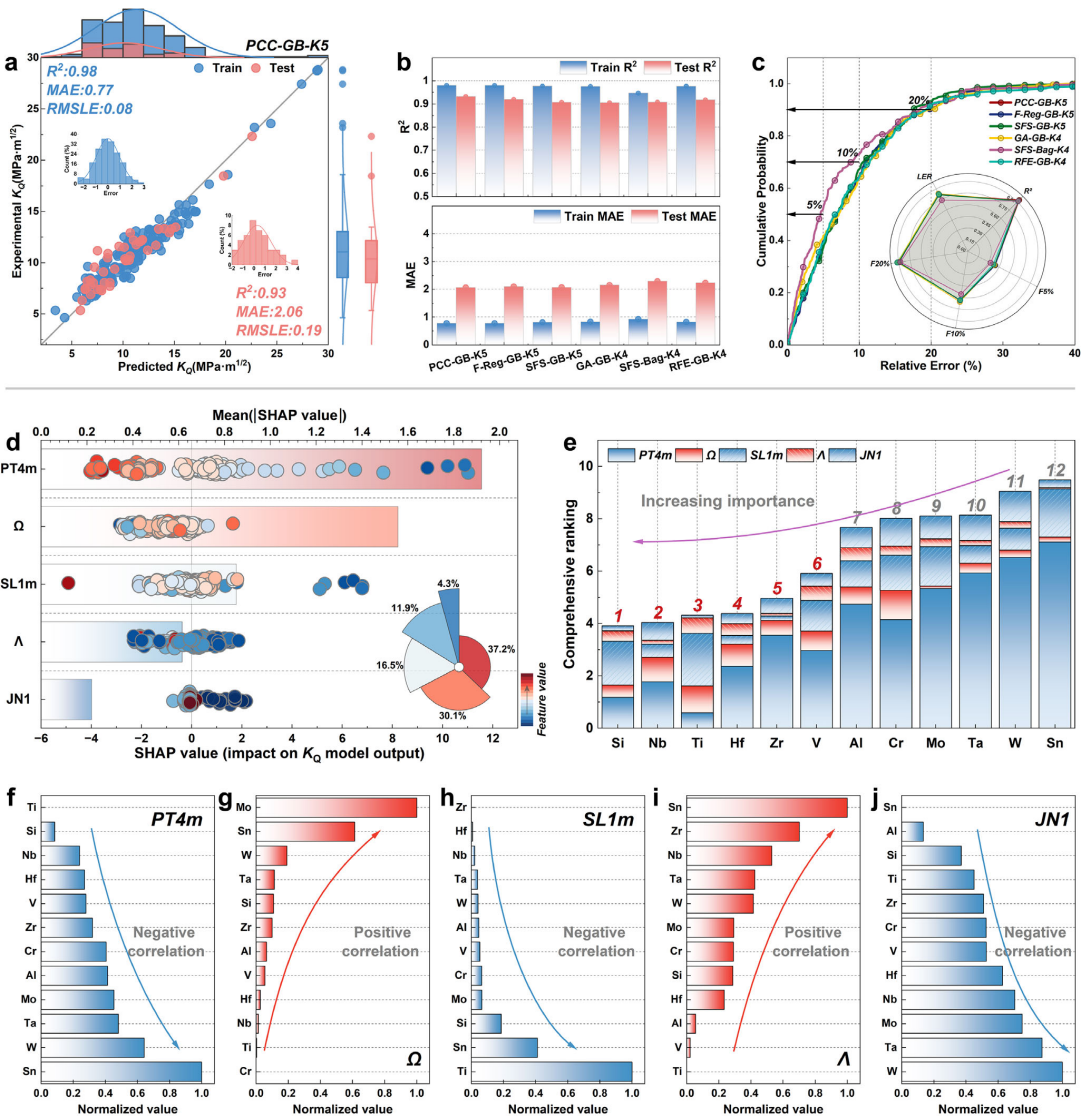
Model prediction performance and SHAP‐based element ranking for Nb─Si alloys. (a) Prediction results of PCC‐GB‐K5 model; (b) R^2^ and MAE comparison of six models; (c) Cumulative probability distribution of relative prediction errors; (d) SHAP values of five key features; (e) Comprehensive element importance ranking from all features; (f–j) Element ranking for individual features: (f) PT4m, (g) Ω, (h) SL1m, (i) Λ, (j) JN1.

To select optimal prediction models, 9 feature selection methods and 16 machine learning models were tested with feature numbers 4, 5, 6, and 7, totaling 576 configurations (9 × 16 × 4). After evaluation using R^2^, MAE, and RMSLE metrics, six optimal model combinations were selected: Pearson correlation coefficient‐based gradient boosting with 5 features (PCC‐GB‐K5), F‐regression‐based gradient boosting with 5 features (F‐Reg‐GB‐K5), sequential forward selection‐based gradient boosting with 5 features (SFS‐GB‐K5), genetic algorithm‐based gradient boosting with 4 features (GA‐GB‐K4), sequential forward selection‐based bagging with 4 features (SFS‐Bag‐K4), and recursive feature elimination‐based gradient boosting with 4 features (RFE‐GB‐K4), as shown in Figure 3a and Figure S1. Details of methods, models, and evaluation results are provided in Supporting Information.

The prediction results show R^2^ values above 0.95 for training sets and 0.91–0.93 for test sets. MAE values range from 0.77 to 2.33, and RMSE values are between 0.08 and 0.22. Figure 3b compares R^2^ and MAE indicators for various models, with RFE‐GB‐K4 performing best on test sets. The cumulative probability distribution curves in Figure 3c show that relative errors for most models concentrate within 10%, with over 90% of prediction results having relative errors less than 20%. To calculate these distributions, we computed relative errors as |(*Exp*—*Pred*)/*Exp*| × 100% for each prediction, removed errors above 50% to focus on the main distribution, and sorted the remaining values in ascending order. Cumulative probabilities were calculated as *P*(*E* ≤ *e*) = *r*/*n*, where *r* is the rank of the error value, and *n* is the total sample count. Residual analysis confirms no systematic bias across the full *K*_Q_ prediction range, with all residuals within ±2σ bounds and Shapiro–Wilk tests supporting normality for both training (p = 0.81) and test sets (p = 0.83); full diagnostics are given in Figure S2. Prediction intervals were further quantified by bootstrapped ensembles (N = 500) and quantile regression, yielding mean uncertainties of approximately 1.2 and 2.2 MPa·m^1/2^, respectively; results are provided in Figures S3 and S4 and Section S1.

To understand how alloy composition affects properties and can guide alloy design, we performed an interpretability analysis on the PCC‐GB‐K5 model. Figure S5a shows the SHAP value distribution patterns for all samples covering the complete training dataset across five key features (PT4m, Ω, SL1m, Λ, JN1), with colors from blue to red representing unfavorable to favorable contributions to *K*_Q,_ respectively. PT4m and Ω features increase *K*_Q_, while the JN1 feature primarily reduces it. The specific meaning of each feature is shown in Table S6. Figure S5b displays the relative importance of five features, with PT4m ranking first at 37.2% contribution, Ω at 30.1%, and SL1m, Λ, and JN1 accounting for 16.5%, 11.9%, and 4.3%, respectively, indicating PT4m and Ω as dominant features affecting *K*_Q_ that should be prioritized in alloy design. Figure S5c–e show SHAP value vs. feature value correlation scatter plots for PT4m, SL1m, and Λ, respectively: the favorable range for PT4m is 24–28, while the 29–32 range should be avoided; SL1m shows both positive and negative SHAP values distributed throughout the entire interval, indicating no clear optimal range; the beneficial range for Λ is 0.1–0.2. These quantitative design windows provide control directions for alloy composition design. The SHAP waterfall plot in Figure S5f uses specific samples as examples, detailing step‐by‐step contribution decomposition processes from model baseline prediction value (12.08 MPa·m^1/2^) to final output value, intuitively illustrating how to maximize *K*_Q_ through synergistic control of multiple feature parameters.

Based on SHAP analysis, we calculated element importance by weighting the 5 key features in the PCC‐GB‐K5 model (process in Section S2. The normalized alloy factor analysis and key alloy factor results are provided in Figure S6 and Table S7, respectively. The element importance ranking is shown in Figure 3d–j. Figure 3e shows element scores affecting *K*_Q_: Si(3.914), Nb(4.040), Ti(4.315), Hf(4.380), Zr(4.966), V(5.911). Lower values indicate a higher ranking. Considering the high cost of the Hf element, based on the balance between importance and cost, Si, Nb, Ti, Zr, and V were selected as candidate elements.

### 2.4 Partial Dependence Plot Analysis for Feature Optimization

**FIGURE 4:**
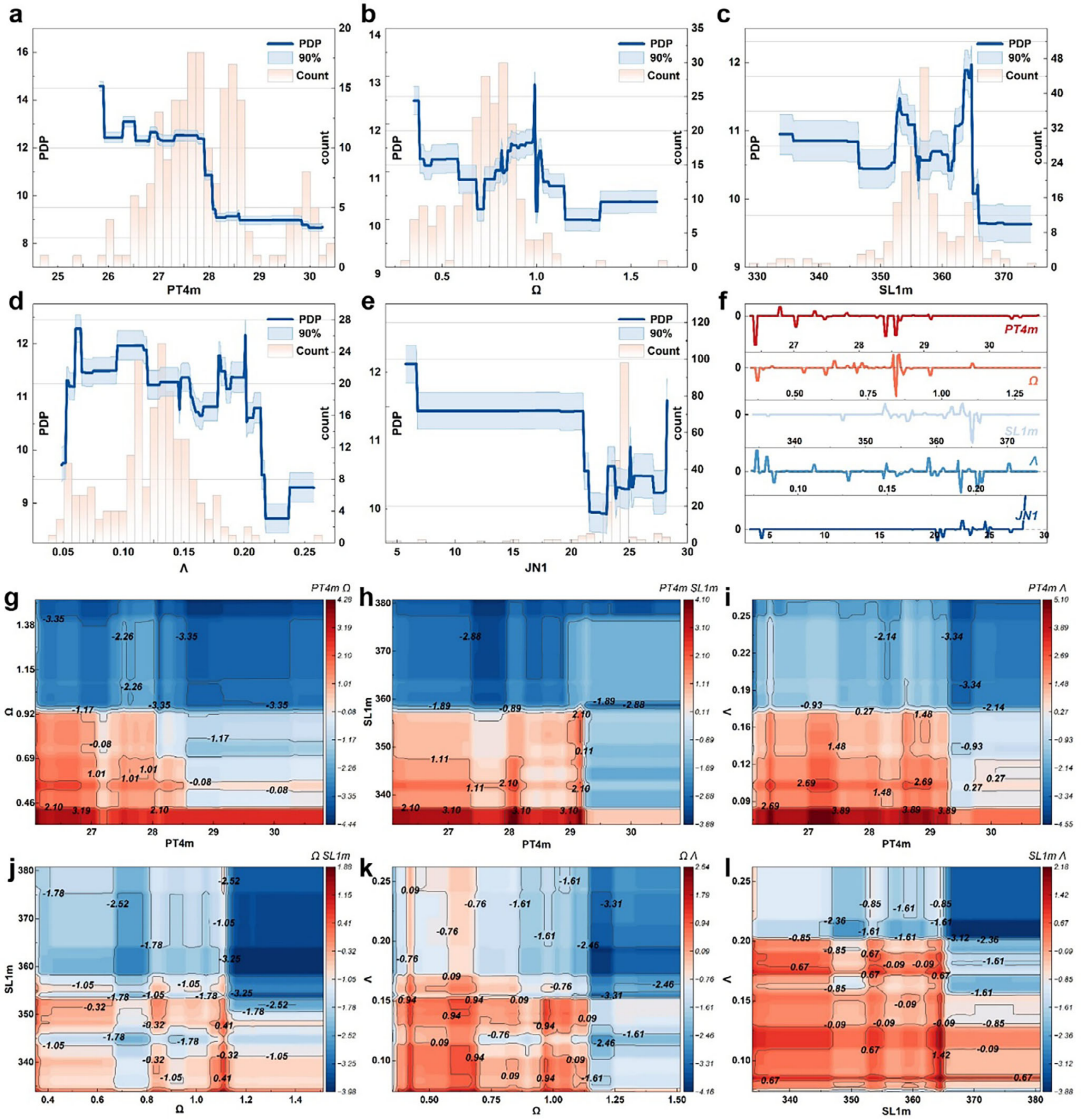
Feature marginal effects and interaction effects based on PDP analysis. (a–e) One‐dimensional PDP analysis; (f) PDP‐gradient values for 5 features; (g–l) Two‐dimensional PDP interaction effect heatmaps for 6 feature pairs.

The one‐dimensional PDP analysis in Figure 4a–e reveals nonlinear influence patterns of five key features on *K*_Q_ prediction, determining optimal parameter ranges for *K*_Q_ optimization. The PT4m feature shows the maximum *K*_Q_ contribution (PDP value 17) in the 26–27 range. Its effect turns negative beyond 28 (declining to 12) and stabilizes at 8–9 beyond 29, revealing a pronounced threshold behavior. The Ω feature provides optimal *K*_Q_ contribution (peak value 13) within the 0.8–1.0 interval, establishing the critical design window for *K*_Q_ optimization. The SL1m feature shows the most favorable impact on *K*_Q_ (peak value 11) in the 345–365 range, displaying a multi‐peak pattern. The Λ feature produces maximum positive effects on *K*_Q_ (peak values 12 and 11) in the 0.06–0.08 and 0.12–0.15 ranges, respectively, presenting dual‐peak characteristics. The JN1 feature demonstrates the most favorable impact on *K*_Q_ (high PDP value 13) in the negative value range, while positive values are detrimental to *K*_Q_ (declining to 10), showing a stepwise pattern. Figure 4f indicates that PT4m and JN1 exhibit the most pronounced gradient changes near critical turning points, confirming their strong sensitivity characteristics for *K*_Q_ regulation. The two‐dimensional PDP analysis covers all ten pairwise combinations of the five key features; the six interactions discussed above are shown in Figure 4g–l, and the remaining four are provided in Figure S7.

The two‐dimensional PDP interaction analysis in Figure 4g–l elucidates synergistic and antagonistic mechanisms between features affecting *K*_Q_. The PT4m‐Ω interaction reveals that high PT4m‐low Ω combinations constitute the optimal *K*_Q_ region (PDP value 1.31), while low PT4m‐high Ω combinations represent the unfavorable *K*_Q_ region (PDP value −4.23). The PT4m‐SL1m interaction presents a diagonal synergistic pattern, with dual high‐value combinations favoring *K*_Q_ enhancement. The PT4m‐Λ interaction identifies the *K*_Q_ optimization combination window of PT4m 28–29 paired with Λ 0.25–0.30. These analyses provide explicit strategies for achieving high *K*_Q_ in Nb─Si alloy design: employing core combinations of high PT4m‐low Ω, high PT4m‐high SL1m, and moderate‐high PT4m‐high Λ, while avoiding low PT4m pairings to ensure *K*_Q_ maximization.

### 2.5 Experimental Validation and Microstructural Analysis

To test the accuracy of our trained machine learning models, we conducted targeted validation focusing on the key feature‐property relationships identified through the interpretability analysis of Sections 2.3 and 2.4. The SHAP analysis showed Si as the most important element affecting *K*_Q_ (importance score 3.914), ranking higher than Nb (4.040) and Ti (4.315). The PDP analysis showed the optimal operating ranges for key features, including PT4m, Ω, and SL1m, that control fracture toughness behavior.

Based on these results from SHAP and PDP, we selected five Nb─Ti─Si─Zr─V alloy compositions (samples #1‐#5) that form a Si‐gradient series with Si contents of 15, 12, 9, 6, and 3 at.%, while maintaining equal contents of Nb and Ti and fixed concentrations of Zr (18 at.%) and V (2 at.%). As shown in Table S8, this composition series tests the importance of Si element on *K*_Q_ as per the SHAP analysis. Furthermore, with PT4m values from 22.88 to 26.216, Ω values from 0.808 to 4.297, and SL1m values from 321.199 to 348.864, we cover the critical feature ranges identified by PDP. This allows us to test the ability of the model to predict *K*_Q_ across different feature combinations.

The five alloys were synthesized by arc melting using high‐purity raw materials and characterized through fracture toughness testing, microstructural analysis, and mechanical property evaluation to compare predicted and experimental *K*_Q_ values.

**FIGURE 5:**
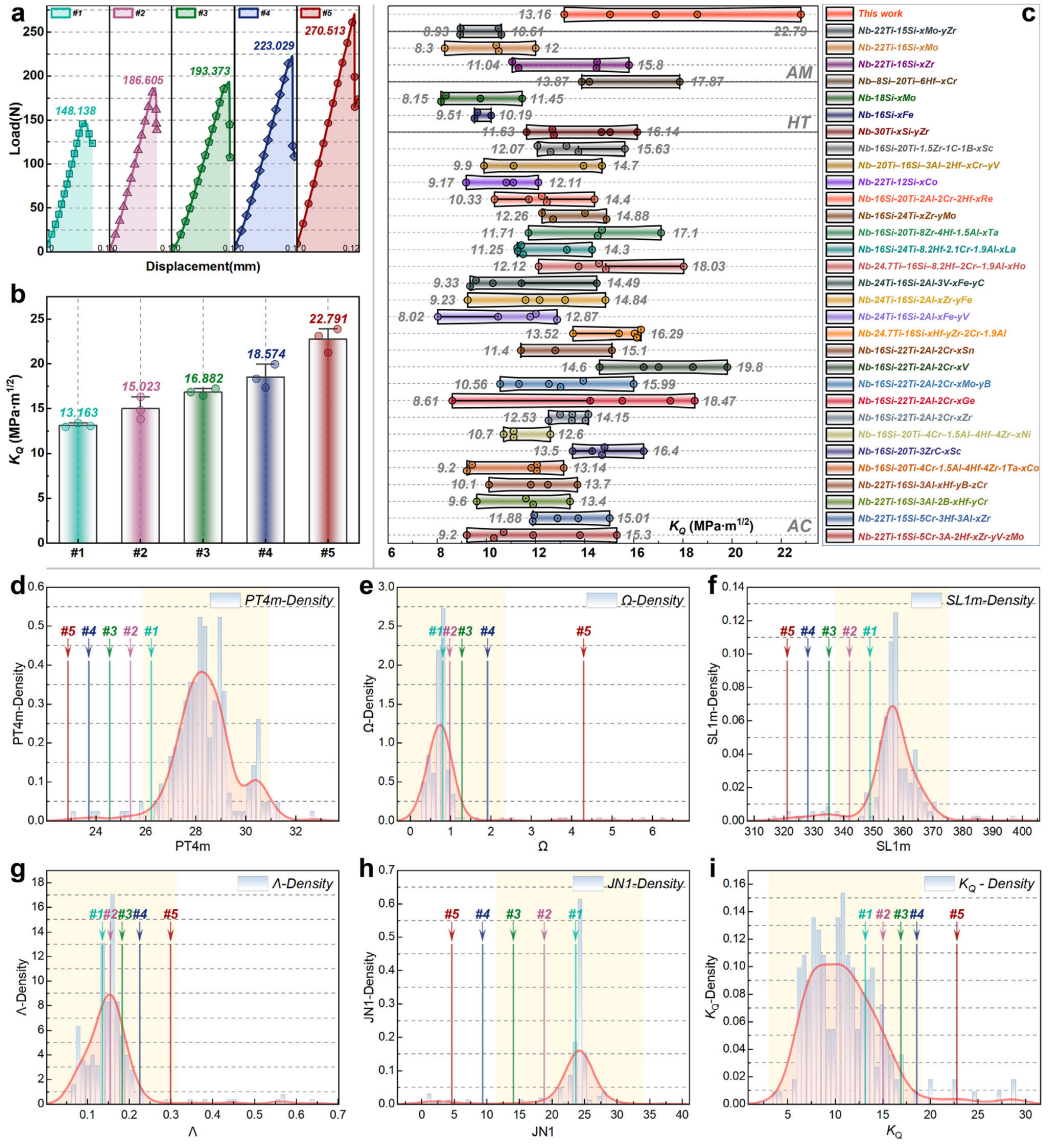
*K*_Q_ characterization and model validation of five alloy samples. (a) Load‐displacement curves; (b) *K*_Q_ values; (c) Comparison with *K*_Q_ of alloys reported in literature: AC (as‐cast), HT (heat‐treated), AM (additive manufacturing) [16, 17, 18, 19, 20, 21, 22, 23, 24, 25, 26, 27, 28, 29, 30, 31, 32, 33, 34, 35, 36, 37, 38, 39, 40, 41, 42, 43, 44, 45]; (d–i) Distribution of 5 samples across feature values and target property: (d) PT4m, (e) Ω, (f) SL1m, (g) Λ, (h) JN1, (i) *K*_Q_.

Figure 5 displays *K*_Q_ test results and microscopic fracture mechanism analysis for five validation alloy samples. Figure 5a,b shows load‐displacement curves from three‐point bending *K*_Q_ tests; sample #5 demonstrates the highest load‐bearing capacity with a load reaching 270.513N. Figure 5b *K*_Q_ test results are highly consistent with load‐displacement curve trends, with sample #5 achieving the highest *K*_Q_ value of 22.791 MPa·m^1/2^, significantly superior to other samples. Figure 5c compares *K*_Q_ results from this study with literature data, categorized by preparation processes into as‐cast (AC), heat‐treated (HT), and additive manufacturing (AM). The optimized alloys developed in this study demonstrate competitive *K*_Q_ levels under all preparation conditions, particularly sample #5, reaching advanced levels among similar materials under as‐cast conditions, proving the effectiveness of the alloy composition optimization strategy.

Figure 5d–i shows the distribution of five synthesized samples in feature space and target properties, where the yellow regions represent the ±2*σ* range of training data. As Si content decreased from sample #1 to #5, sample positions systematically moved toward and beyond the ±2*σ* boundaries, showing that the experimental design effectively explored the feature space identified by interpretability analysis. The comparison in Table S9 indicates that prediction errors for all models are controlled within 7%, with sample #5 exceeding the upper ±2*σ* boundary for *K*_Q_, confirming successful discovery of alloy compositions with performance beyond the training data range. This prediction accuracy represents a substantial improvement over the constrained mixture model (Section S3, which yields a mean error of 27.13% on the same five alloys (Figures S8 and S9 and Table S10). This gap reflects the ML model's ability to capture nonlinear composition‐microstructure‐property relationships beyond simple geometric constraints. Quantitative extrapolation risk analysis in the five‐dimensional feature space further shows that alloys #1‐#3 lie within the training distribution while alloys #4 and #5 extend into the high‐risk zone (Mahalanobis distance > 5.0), confirming that the experimental design achieved controlled exploration beyond the training data manifold (Figure S10).

**FIGURE 6:**
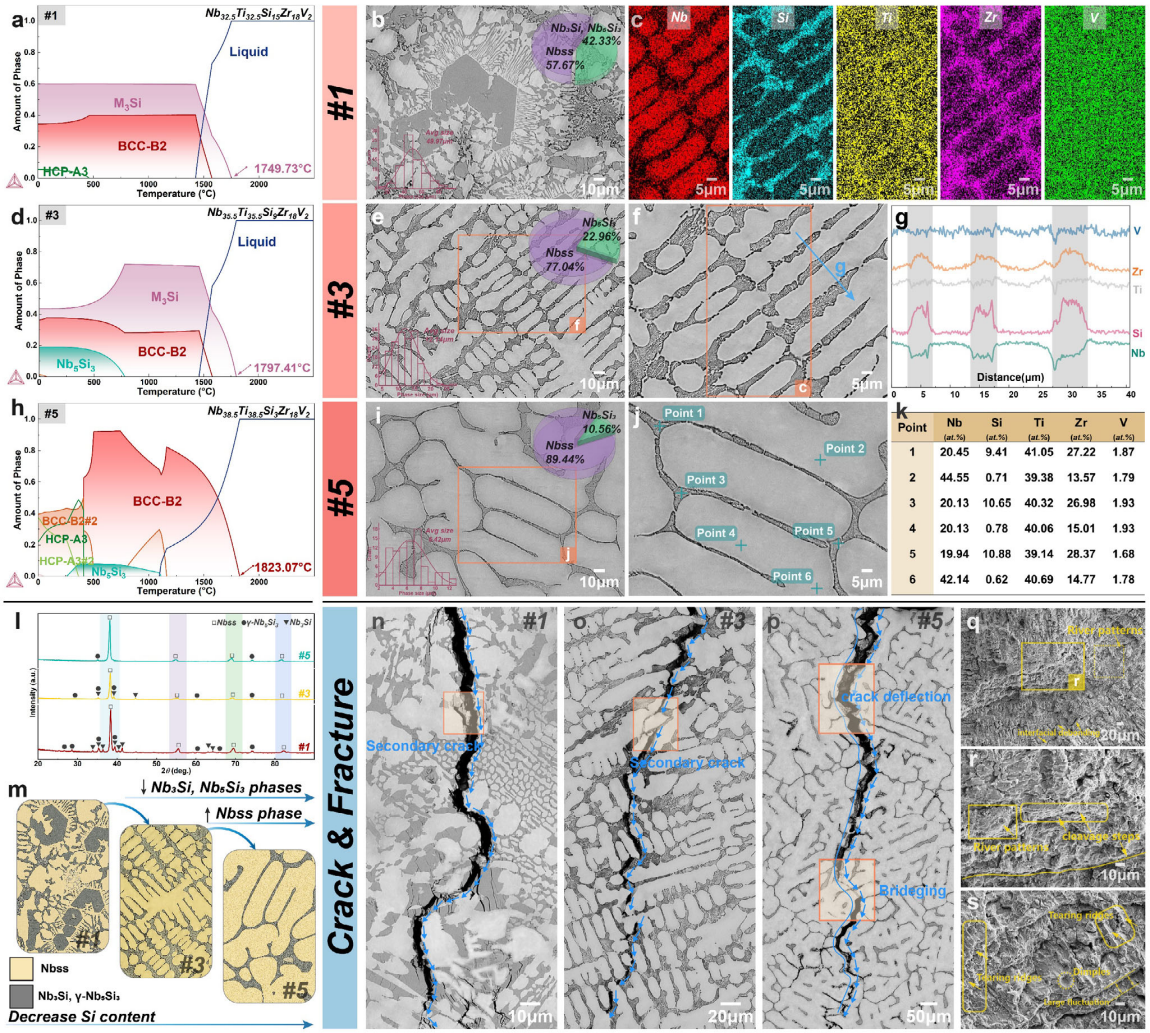
Phase composition characterization, thermodynamic analysis, and microstructural features of the validation alloy samples. (a, d, h) Thermodynamic phase diagrams for samples #1, #3, #5; (b, e, f, i, j) SEM images for samples #1, #3, #5; (c) EDS elemental maps for sample #3; (g) EDS line scanning across Nbss/silicide interface for sample #3; (k) EDS point composition analysis for sample #5; (l) XRD diffraction patterns for samples #1, #3, #5; (m) Schematic diagram of microstructural evolution mechanism; (n–p) SEM images of crack propagation for samples #1, #3, #5; (q–s) Fracture surface morphology of sample #5.

Figure 6 presents phase composition characterization, thermodynamic analysis, and microstructural features of the validation alloy samples. Figure 6a,d,h displays phase equilibrium evolution processes from room temperature to 2500°C for samples #1, #3, and #5 through Thermo‐Calc thermodynamic calculations, showing that all alloys are dominated by BCC‐B2 structured Nbss matrix phase at low temperatures, with sequential appearance of Nb_3_Si phase and Nb_5_Si_3_ phase precipitation and dissolution transformations as temperature increases. Liquidus temperatures differ significantly among samples: sample #1 exhibits the highest high‐temperature stability (1748.73°C), sample #3 has a liquidus temperature of 1797.41°C, and sample #5 reaches 1823.07°C. Figure 6l shows XRD diffraction patterns, with the main phase compositions of all samples being Nbss phase and Nb_3_Si, Nb_5_Si_3_ silicide phases. High Ti content favors enhanced high‐temperature phase stability, while appropriate Si content ensures the stable existence of the Nb_5_Si_3_ strengthening phase. The thermodynamic calculation results agree with XRD experimental observations.

Figure 6b,e,f,i,j respectively show SEM microstructural images for samples #1, #3, #5. Average precipitate sizes sequentially decrease from 49.97 µm in sample #1 to 28.6 µm in sample #3, and finally 15.8 µm in sample #5. Volume fraction of ductile Nbss phase progressively increases from 62% in sample #1% to 78% in sample #5. Correspondingly, the total volume fraction of brittle silicide phases (mainly including Nb_5_Si_3_ and Nb_3_Si phases) decreases from 38% to 22%. This phase composition transformation is the main reason for material toughness improvement, as the ductile Nbss phase possesses better plastic deformation and crack propagation resistance [46].

EDS analysis in Figure 6c,g,k systematically reveals distribution patterns and phase composition characteristics of major elements. Area scanning results show Nb element uniformly distributed in matrix phase (red regions), Si element concentrated in precipitate phases (cyan regions), while Ti and V elements are relatively uniformly distributed in the matrix. Line scanning quantitative analysis confirms significant element concentration gradients, with Nb content decreasing from 92 at.% in the matrix phase to 68 at.% in the precipitate phase, while Si content shows opposite trends, jumping from 5 at.% in the matrix phase to 32 at.% in the precipitate phase. Point composition analysis further confirms phase identification accuracy, with matrix phase regions mainly being Nbss phase and precipitate phase regions corresponding to silicide phases. Figure 6m is a mechanism schematic diagram, showing the phase composition transformation process from brittle Nb_3_Si, Nb_5_Si_3_ silicide phase dominance toward ductile Nbss phase dominance with decreasing Si content.

SEM microstructural analysis of crack propagation in Figure 6n–p shows that secondary cracks are observed in samples #1 and #3, while sample #5 exhibits crack bridging phenomena and crack deflection that effectively reduce crack propagation energy and enhance the *K*_Q_ of the alloy [47].

Figure 6q,r shows fracture surface morphology of sample #5, with Nbss‐rich regions displaying characteristic ductile dimples and silicide‐rich regions showing brittle features. Figure 6s displays mixed fracture characteristics where ductile tearing transitions to cleavage facets at phase boundaries.

Comprehensive mechanical property characterization is provided in Figures S11–S13. The results demonstrate that sample #5 achieves an excellent strength‐toughness balance with compressive strength of 1381.37 MPa and strain of 50%, while maintaining the breakthrough fracture toughness of 22.791 MPa·m^1/2^. Strengthening mechanism analysis reveals that solid solution strengthening is the dominant mechanism, contributing 68%–84% of the total strength across all samples, indicating that the excellent mechanical properties are achieved primarily through intrinsic solid solution effects rather than relying on brittle precipitate strengthening (process in Section S4). This explains the simultaneous achievement of high strength and high toughness in sample #5, and performance comparison confirms that the optimized compositions exhibit superior strength‐plasticity‐toughness combinations compared to traditional Nb‐based, Ti‐based, and refractory high‐entropy alloys [48, 49, 50, 51, 52, 53, 54, 55, 56, 57, 58, 59, 60, 61, 62, 63, 64, 65, 66, 67, 68, 69, 70, 71, 72, 73, 74, 75, 76, 77, 78].

### 2.6 Model Validation and Formula Fitting Analysis

To facilitate engineering applications, we fitted a formula to the machine learning predictions. Figure S14 shows the analysis results for six machine learning models. During the fitting process, various mathematical descriptors were used for the five key parameters (PT4m, Ω, SL1m, Λ, JN1), including ratios (x1/x2), products (x1 × x2), square roots (√x), squares (x^2^), and inverse terms (1/x). The fitting employed Lasso regression with alpha values ranging from 0.001 to 0.5, determined through fivefold cross‐validation. Bayesian optimization used Gaussian process minimization with 50 function evaluations to optimize hyperparameters.

All models fit the formula with R^2^ values exceeding 0.90. Of these, the PCC‐GB‐K5 model fits the expression $K_{Q}=12.081-12.12\cdot \Omega^{2}-8.97\cdot \frac{\mathrm{JN}1}{\mathrm{PT}4m}$ (R^2^ = 0.9169), highlighting Ω quadratic term effects and negative regulatory effects of JN1; F‐Reg‐GB‐K5 model fits to $K_{Q}=12.081+8.7\cdot \frac{\Lambda }{\mathrm{PT}4m}+7.27\cdot \exp(\Omega +\Lambda )$ (R^2^ = 0.917), reflecting linear contributions of Λ and PT4m ratios and exponential effects of Ω, Λ combinations; SFS‐GB‐K5 model fits to $K_{Q}=12.081+16.40\cdot \sqrt{\Omega \cdot \mathrm{PT}4m}$ (R^2^ = 0.9306), achieving the highest fitting accuracy through only geometric mean interaction terms of Ω and PT4m.

These expressions reveal three key findings: (1) High Si content reduces *K*_Q_, as shown by negative coefficients of JN1‐related terms. This agrees with experimental results where brittle Nb_3_Si and Nb_5_Si_3_ phases dominate at high Si concentrations, reducing the ductile Nbss phase fraction. The ratio form (JN1/PT4m) indicates that Si effects are affected by melting enthalpy. (2) The different functional forms (quadratic, exponential, and square root terms) show nonlinear relationships between thermodynamic parameters (Ω and Λ) and melting enthalpy (PT4m). (3) The baseline value (12.081 MPa·m^1/2^) appears in all models and represents the baseline fracture toughness of the Nb─Si system.

## 3 Conclusions

We establish a machine learning workflow for Nb─Si alloys. The main conclusions are as follows:

1. A data‐driven workflow encompassing database construction, feature engineering, model construction, interpretability analysis, and experimental validation is established.
2. A three‐step progressive feature screening strategy was implemented, identifying key factors including PT4m, Ω, SL1m, Λ, and JN1 from 139 initial features. The gradient boosting models had prediction accuracy with R^2^ of 0.91–0.93 and prediction error <7%.
3. A SHAP analysis on the Nb─Si alloy system yielded PT4m (contribution rate 37.2%) and Ω (contribution rate 30.1%) as key features affecting *K*_Q_. A PDP analysis elucidated nonlinear interaction effects between features. Formulas were also fitted and compared to the machine learning predictions (R^2^>0.90).
4. Five validation alloys (samples #1‐#5) were synthesized to test model predictions. Sample #5 achieved *K*_Q_ of 22.791 MPa·m^1/2^ and compressive strength of 1381.37 MPa, exceeding 20 MPa·m^1/2^ for as‐cast Nb─Si alloys.
5. The microscopic mechanisms of performance improvement were identified: phase composition transformation from brittle Nb_3_Si and Nb_5_Si_3_ silicide phases toward ductile Nbss phase. Quantitative analysis indicated solid solution strengthening as the dominant mechanism (68%–84%), achieving excellent strength‐toughness balance.

## 4 Experimental Section

### 4.1 Data Collection and Database Construction

We constructed a database containing 599 Nb─Si alloy data points, covering alloy composition, physicochemical properties, and mechanical performance data. Figure S15 shows the frequency distribution of elements in the Nb─Si alloy database. Results show that Nb, as the matrix element, appears with 100% frequency. Transition metal element Ti shows a high usage frequency of 87%, indicating its important role in Nb─Si alloy design. Elements Zr and V also exhibit certain usage frequencies (38% and 8.7%). Rare earth elements and alkali metals show extremely low usage frequencies, indicating that in Nb─Si alloy systems, alloying elements are primarily concentrated within transition metals and some main group elements. Based on these elemental distribution patterns and focusing on as‐cast conditions, we ultimately constructed a database subset containing 216 as‐cast data points.

The compositional distribution and target property analysis of the Nb─Si alloy database are presented in Figure S16. Figure S16a demonstrates the statistical distribution of alloy component numbers, indicating that binary alloys constitute 15%, ternary alloy systems represent the highest proportion at 45%, quaternary alloy systems account for 30%, and complex alloy systems with five or more elements comprise 10%, spanning from simple to complex alloy systems. Figure S16b reveals the *K*_Q_ distribution, with values primarily distributed within the 5–17 MPa·m^1/2^ range, exhibiting a mean of 11.06 MPa·m^1/2^ and normal distribution characteristics. High‐performance samples (*K*_Q_>18MPa·m^1/2^) represent approximately 15% of the dataset, providing high‐performance sample references for model training. The dataset is restricted to arc‐melted samples. Nine arc‐melting processing parameters were extracted from all 216 literature sources and summarized in Tables S11 and S12 and Section S5. Variance decomposition confirms that compositional factors account for ∼96% of total *K*_Q_ variance, with combined processing and measurement contributions estimated at 0.45–0.54 = MPa·m^1/2^ (∼1%–2%; Table S13 and Figure S17). Model predictions are therefore applicable to alloys prepared under arc‐melting conditions consistent with the training dataset, with an estimated uncertainty of ±1.0 MPa·m^1/2^ from residual processing variability.

### 4.2 Feature Engineering

Based on materials physics mechanisms, intrinsic physicochemical features of various elements were extracted. The constructed feature system includes 103 basic features covering: (1) atomic properties (atomic radius, atomic mass, atomic volume, etc.); (2) electronic properties (electron concentration, valence electrons, electronegativity, etc.); (3) thermodynamic properties (melting point, boiling point, formation enthalpy, etc.); (4) physical transport properties (thermal conductivity, electrical conductivity, etc.); (5) mechanical properties (elastic modulus, Poisson's ratio, etc.); (6) structural characteristics (crystal structure, coordination number, etc.). Additionally, 36 domain‐informed features were constructed from the 103 fundamental features based on materials science principles, including toughness criteria, thermodynamic stability, and phase formation theory, totaling 139 candidate features.

We used a three‐step feature screening method to select key features from 139 features. First, Pearson correlation coefficient (PCC) analysis removed redundant features using a threshold of |r| > 0.95. Second, recursive feature elimination (RFE) iteratively removed features by evaluating their contribution to prediction accuracy using Ridge regression, Huber regression, and Random Forest, removing the least important feature in each cycle until reaching 40 features. Third, nine feature selection algorithms evaluated subset sizes from 2 to 15 features to find the best number. Details are in Section 2.2.

### 4.3 Model Construction and Evaluation

The dataset was randomly split into training and test sets with an 8:2 ratio, and this process was repeated 10 times to reduce the impact of randomness from single data splitting. Model performance was evaluated through multiple metrics on both training and test sets, including coefficient of determination (R^2^), mean absolute error (MAE), root mean square error (RMSE), and root mean square logarithmic error (RMSLE). Error was minimized through tenfold cross‐validation.

(1) $$R^{2}=1-\frac{\sum_{i=1}^{n}{\left(Exp_{i}-Pre_{i}\right)}^{2}}{\sum_{i=1}^{n}{\left(Exp_{i}-\bar{Exp}\right)}^{2}}$$

(2) $$MAE=\frac{1}{n}\sum_{i=1}^{n}\left|Exp_{i}-Pre_{i}\right|$$

(3) $$RMSE=\sqrt{\frac{1}{n}\sum_{i=1}^{n}{\left(Exp_{i}-Pre_{i}\right)}^{2}}$$

(4) $$RMSLE=\sqrt{\frac{1}{n}\sum_{i=1}^{n}{\left[\log\left(Exp_{i}+1\right)-\log\left(Pre_{i}+1\right)\right]}^{2}}$$

where *Exp_i_* and *Pre_i_* are experimental and predicted values, respectively, $\bar{Exp}$ is the mean of experimental values, and n is the number of samples.

### 4.4 Interpretability Analysis

The SHAP(ley) method was employed to enhance model interpretability [79]. SHAP is based on the Shapley value concept from game theory, quantifying the marginal contribution of each feature to model predictions. Compared to traditional feature importance methods, SHAP satisfies axiomatic properties including efficiency, symmetry, dummy, and additivity, ensuring the theoretical rigor of analysis results.

For gradient boosting models, TreeExplainer was used to calculate SHAP values:

(5) $$\phi_{i}=\sum_{S\subseteq F\setminus \left\{i\right\}}\frac{\left|S\right|!\left(\left|F\right|-\left|S\right|-1\right)!}{\left|F\right|!}\left[f_{x}\left(S\cup \left\{i\right\}\right)-f_{x}\left(S\right)\right]$$

where *F* is the feature set, *S* is a feature subset not containing feature i, and *f_x_* (*S*) is the model prediction value based on feature subset *S*.

Feature marginal effects and interaction effects on model output were explored through PDP analysis:

(6) $$PDP_{S}\left(x_{S}\right)=E_{x_{C}}\left[f\left(x_{S},x_{C}\right)\right]=\int f\left(x_{S},x_{C}\right)p\left(x_{C}\right)dx_{C}$$

where *x_S_* is the target feature, *x_C_* represents other features, and *p*(*x_C_*) is the marginal distribution of other features. One‐dimensional PDP shows marginal effects of individual features, while two‐dimensional PDP reveals interactions between features.

### 4.5 Alloy Design and Experimental Validation

Based on SHAP and PDP analysis results, five alloy compositions with Si contents of 15, 12, 9, 6, and 3 at.% (samples #1‐#5) were selected to test model predictions.

Samples were synthesized using arc melting, with high‐purity metal raw materials (purity >99.9%) under inert argon. Each sample was remelted at least 7 times to ensure compositional homogeneity. The obtained ingots were cut into standard specimens by wire cutting for performance testing and microstructural characterization. *K*_Q_ testing used three‐point bending, with specimen dimensions 2 × 4 × 20 mm and a pre‐crack length of approximately 2 mm. To ensure result accuracy, three identical specimens underwent *K*_Q_ evaluation. Compression testing was conducted employing a universal testing apparatus (Instron 5569) at a steady loading rate of 0.2 mm/min. Sample dimensions were Φ4 × 6 mm. To ensure result accuracy, three identical specimens underwent compressive evaluation.

Microstructural characterization used an X‐ray diffractometer (XRD, Empyrean) to analyze phase composition, operating between 20°–100° at a scanning speed of 8°/min. A scanning electron microscope (SEM, Merlin Compact) combined with an energy dispersive spectrometer (EDS) was used to analyze microstructure and element distribution.

## Author Contributions

D.C., C.X., and R.C. supervised the project. C.X. and D.C. conceived the idea and designed the methodology. D.C. and J.Y. conducted the investigation and experiments. D.C. performed the formal analysis. C.X. curated the data and created the visualizations. T.L. validated the results. R.C. administered the project, and R.C. and C.X. acquired the funding. D.C. wrote the original draft. C.X., J.Y., Q.W., H.F., S.Y., T.L., and R.C. contributed to the review and editing of the manuscript. All authors participated in the discussion of the results and approved the final version of the manuscript.

## Conflicts of Interest

The authors declare no conflicts of interest.

## Supporting information




**Supporting File 1**: advs75815‐sup‐0001‐SuppMat.docx.

**Supporting File 2**: advs75815‐sup‐0002‐Data1.xlsx.

**Supporting File 3**: advs75815‐sup‐0002‐Data2.xlsx.

## Data Availability

The data that support the findings of this study are available on request from the corresponding author. The data are not publicly available due to privacy or ethical restrictions.
