# Supplementary material for: Interpretable Machine Learning Framework for Nb─Si Based Alloy Design with Enhanced Fracture Toughness
Source: Adv Sci (Weinh). 2026 May 25:e75815. Online ahead of print. doi: 10.1002/advs.75815 (PMC13336009; doi:10.1002/advs.75815)
Supplement: Supplementary file 1 — Supporting File 1: advs75815‐sup‐0001‐SuppMat.docx. [file ADVS-9999-e75815-s002.docx]

**Supplementary Information**

**Interpretable machine learning framework for Nb-Si based alloy design with enhanced fracture toughness**

Dezhi Chen^a,^^b^, Chao Xu ^a*^, Jingyue Yu^a^, Qi Wang^a^, Hongze Fang^a^, Shuo Yin^b^, Turab Lookman^c,d*^, Ruirun Chen^a*^

*^a^, National Key Laboratory for Precision Hot Processing of Metals, Harbin Institute of Technology, 150001, PR China*

*^b^, Department of Mechanical, Manufacturing, and Biomedical Engineering, Trinity College, University of Dublin, Ireland*

*^c^, State Key Laboratory for Mechanical Behavior of Materials, State Key Laboratory of Porous Metal Materials, School of Materials Science and Engineering, Xi’an Jiatong University, Xi’an, China*

*^d^, AiMaterials Research LLC, Santa Fe, NM, 87501, USA*

*Corresponding author: Chao Xu, 22s109243@stu.hit.edu.cn; Turab Lookman, [txl_24@xjtu.edu.cn](mailto:txl_24@xjtu.edu.cn) ; Runrui Chen, [ruirunchen@hit.edu.cn](mailto:ruirunchen@hit.edu.cn)

This PDF file includes:

Figures. S1 to S17

Tables S1 to S13

References

**Figures**


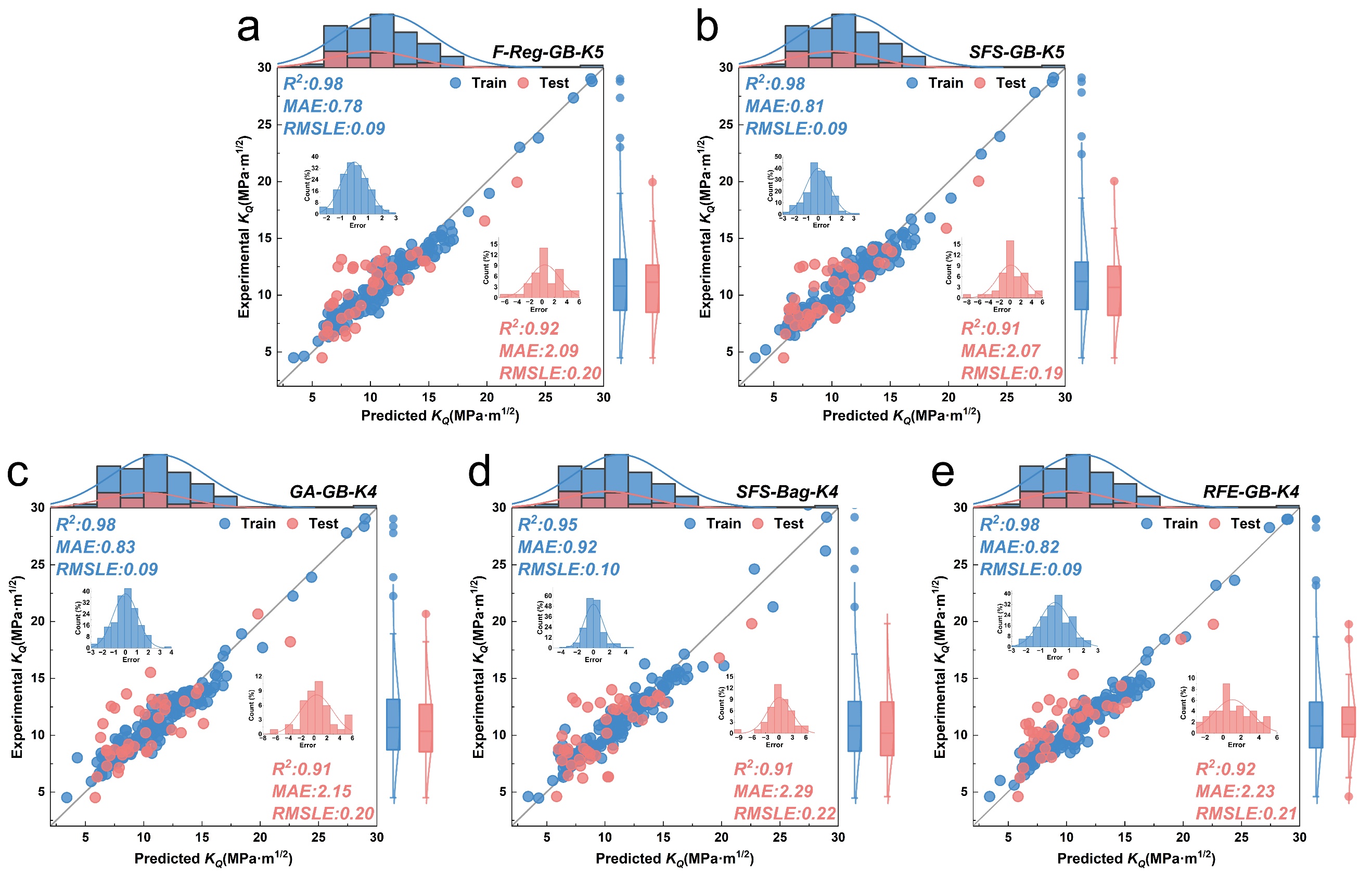


**Figure S1.** *K*_Q_ prediction performance for Nb-Si alloys. (a-f) Results of five models: (a) F-Reg-GB-K5, (b) SFS-GB-K5, (c) GA-GB-K4, (d) SFS-Bag-K4, (e) RFE-GB-K4.


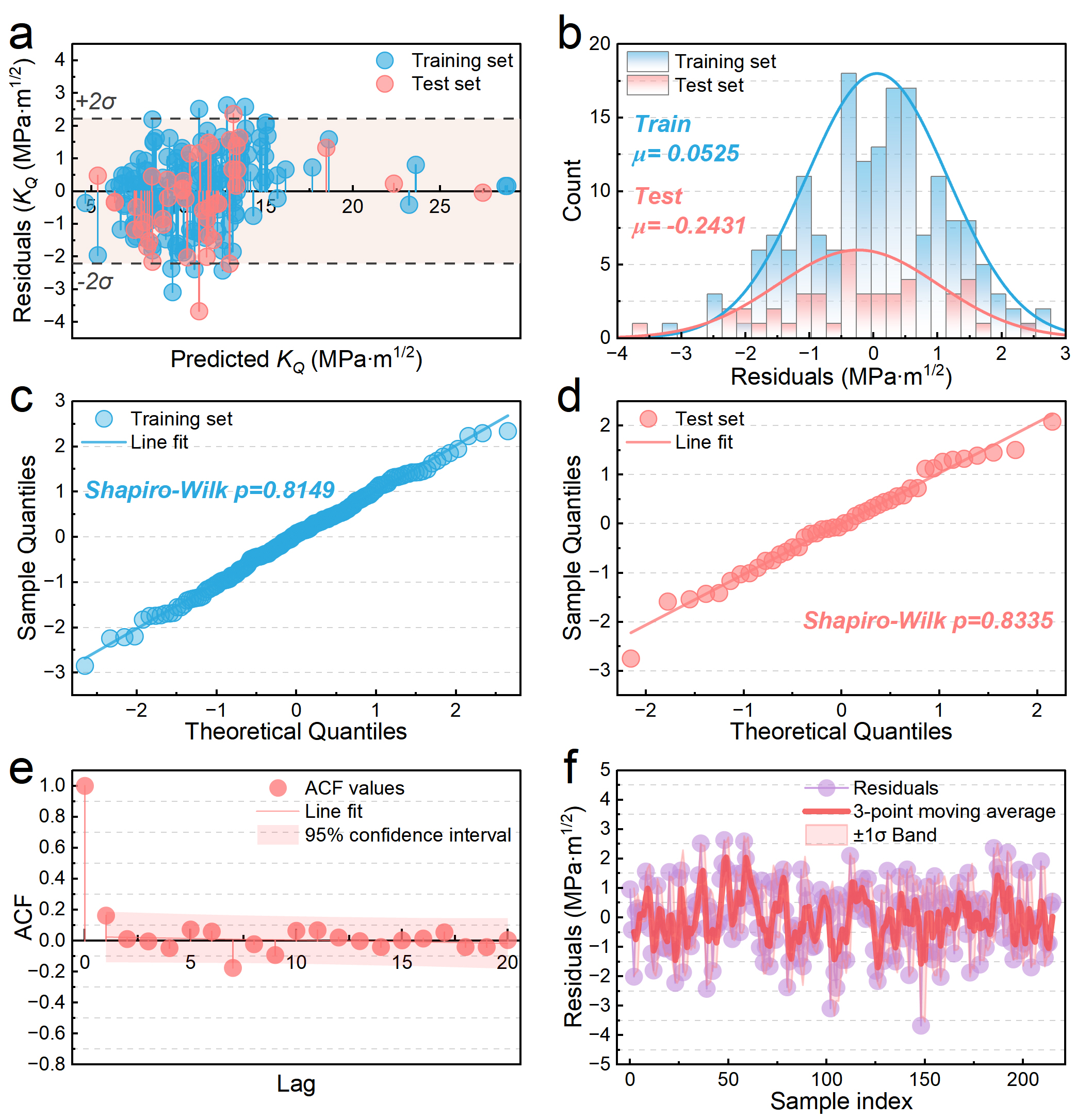


**Figure. S2**. Residual diagnostics for the *K*_Q_ model. (a) residuals vs. predicted values with ±2*σ* bounds; (b) residual histograms for training and test sets; (c) Q-Q plot for the training set; (d) Q-Q plot for the test set; (e) autocorrelation function with 95% confidence interval; (f) residuals versus sample index with 3-point moving average and ±1*σ* bands.


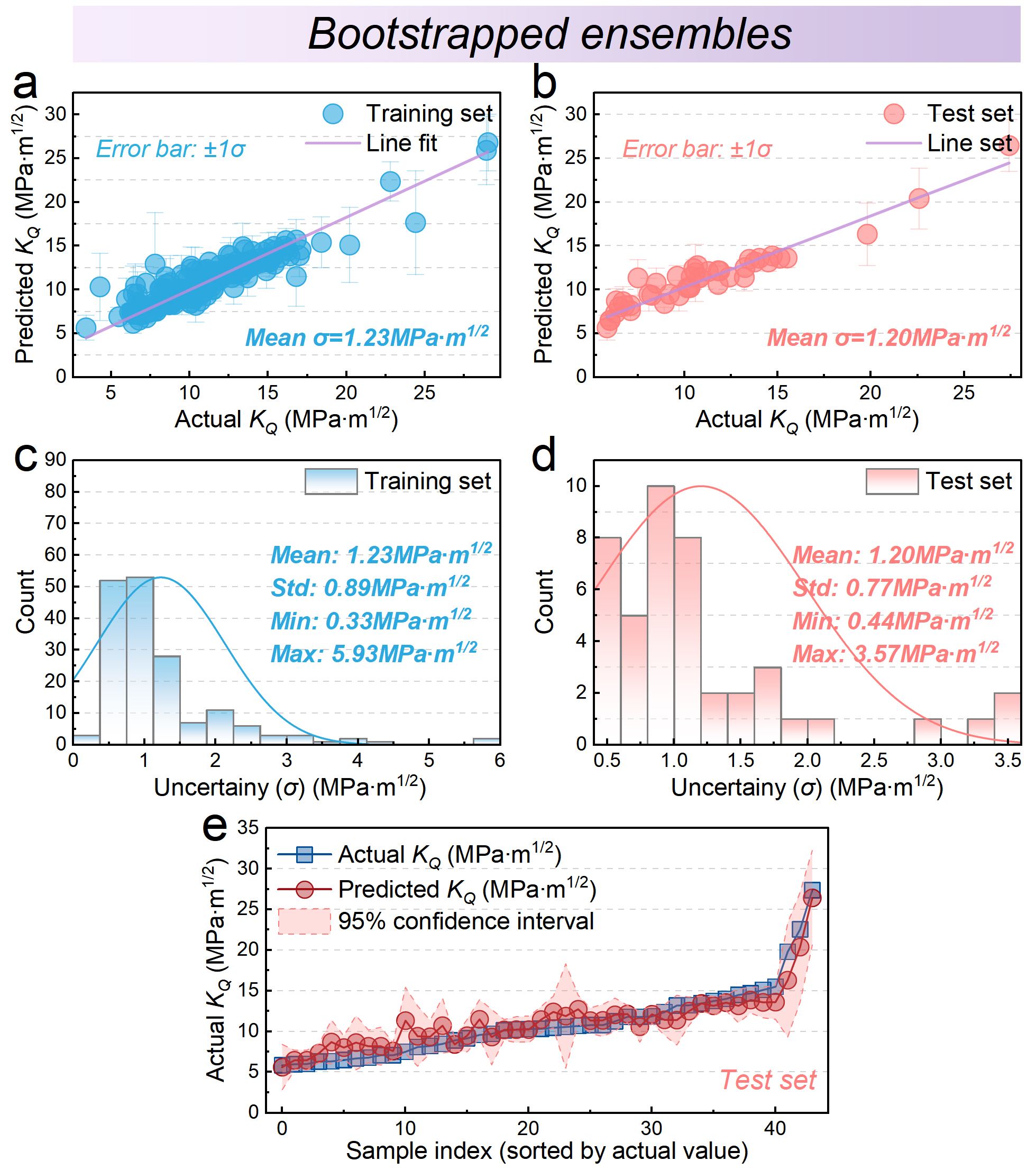


**Figure. S3**. Bootstrap ensemble uncertainty quantification for the *K*_Q_ model. (a) Predicted vs. actual *K*_Q_ for the training set with ±1σ error bars; (b) predicted vs. actual *K*_Q_ for the test set with ±1σ error bars; (c) distribution of prediction uncertainties for the training set; (d) distribution of prediction uncertainties for the test set; (e) test sample predictions with 95% confidence intervals sorted by actual value.


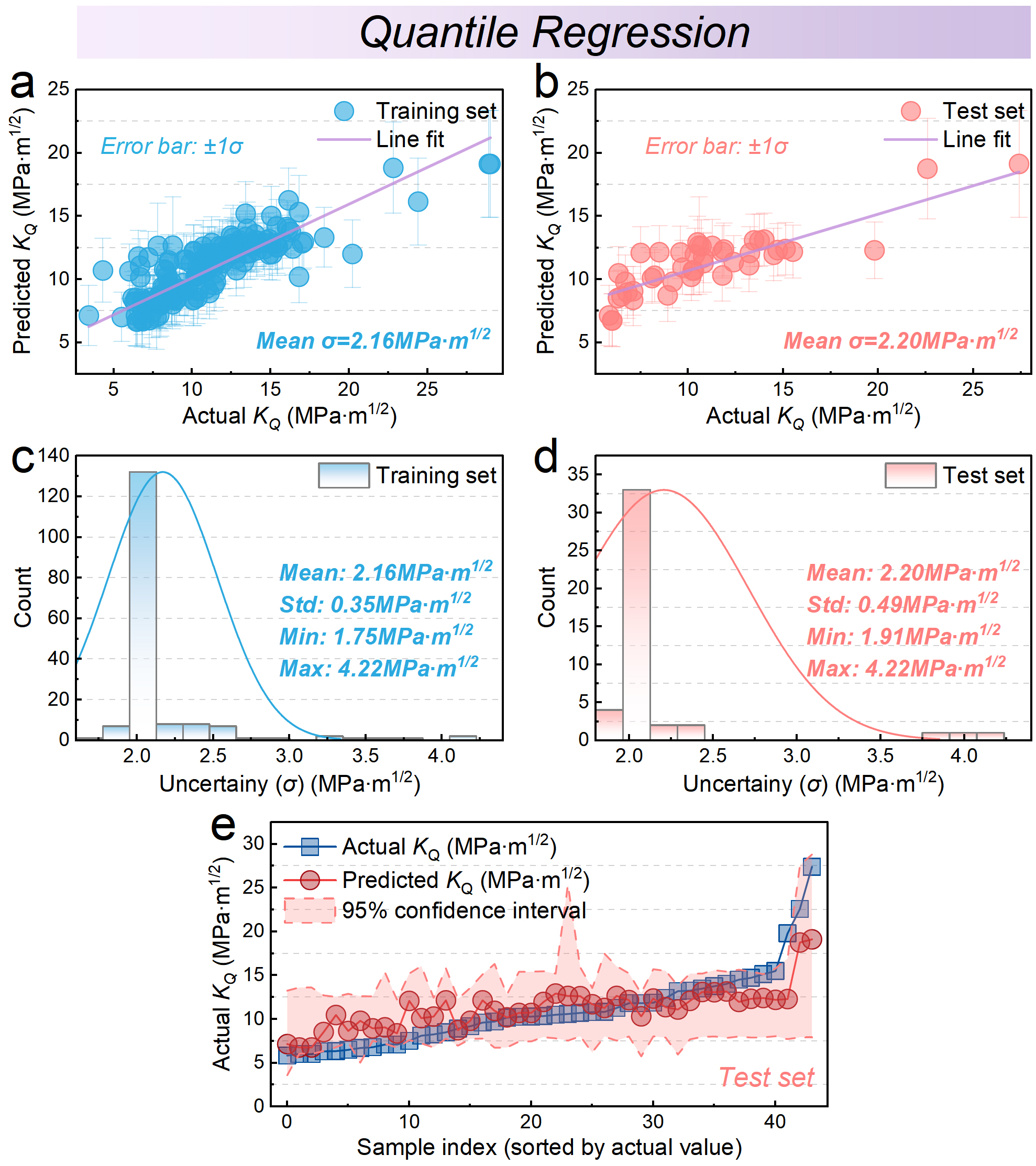


**Figure. S4**. Quantile regression uncertainty quantification for the *K*_Q_ model. (a) Predicted vs. actual *K*_Q_ for the training set with ±1σ error bars; (b) predicted vs. actual *K*_Q_ for the test set with ±1σ error bars; (c) distribution of prediction uncertainties for the training set; (d) distribution of prediction uncertainties for the test set; (e) test sample predictions with 95% confidence intervals sorted by actual value.


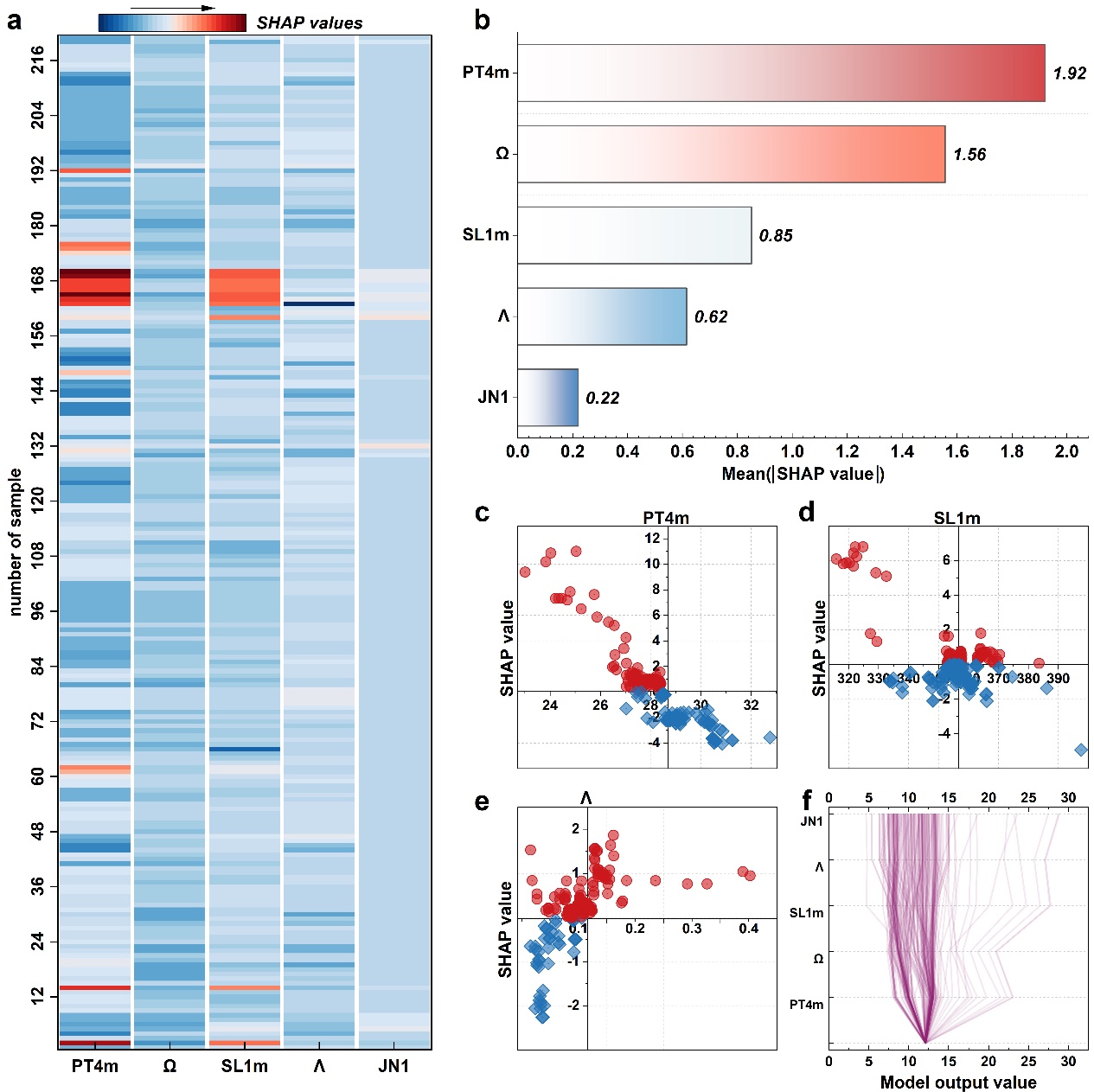


**Figure S5.** SHAP analysis of PCC-GB-K5 model. (a) SHAP value distribution heatmap; (b) Feature importance bar chart and proportion pie chart; (c-e) The dependency relationships of feature and SHAP value: (c) PT4m, (d) SL1m, (e) Λ; (f) SHAP waterfall plot.


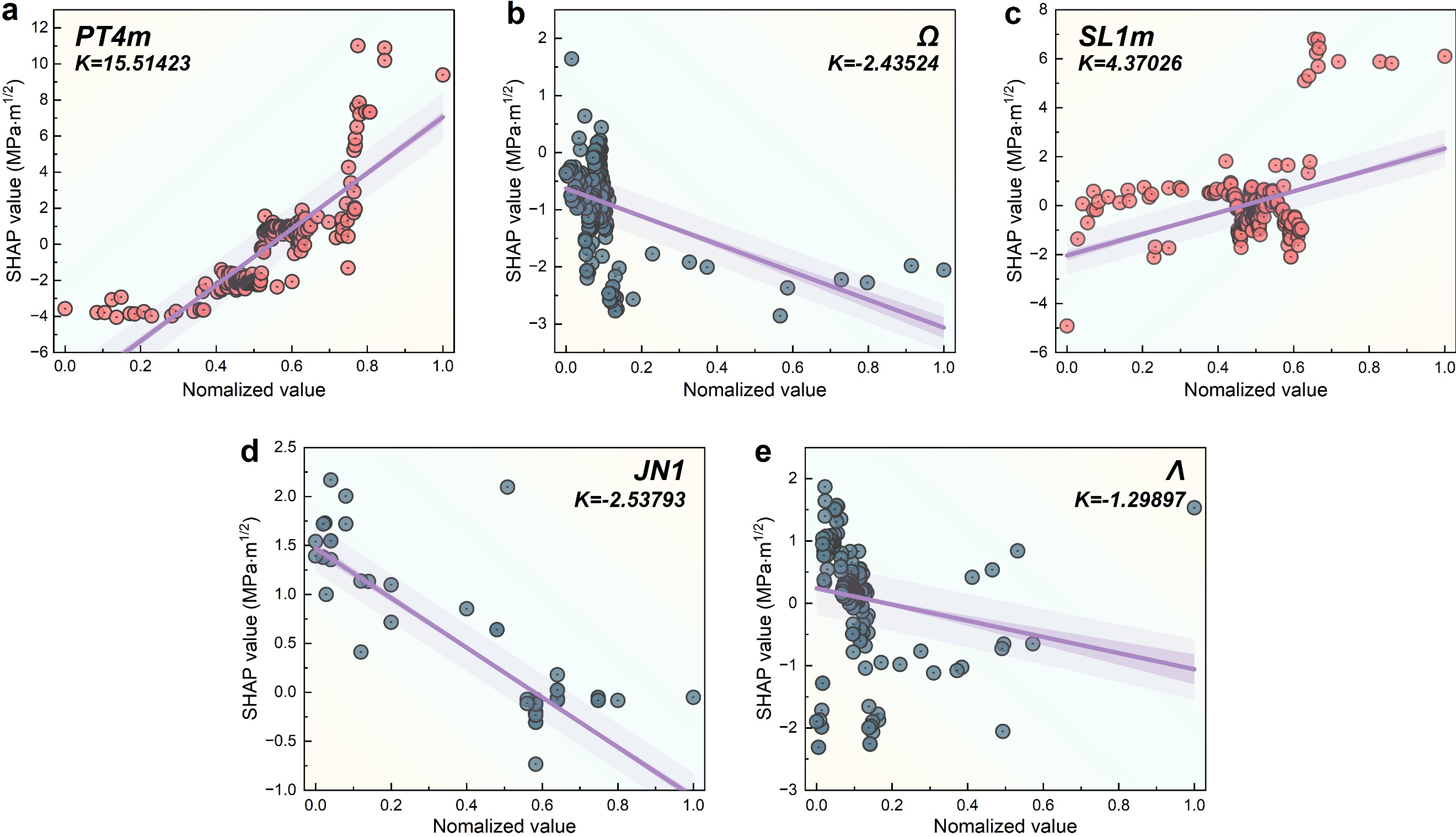


**Figure S6.** Normalized alloy factors and corresponding SHAP values. (a) PT4m, (b) Ω, (c) SL1m, (d) Λ, (e) JN1.


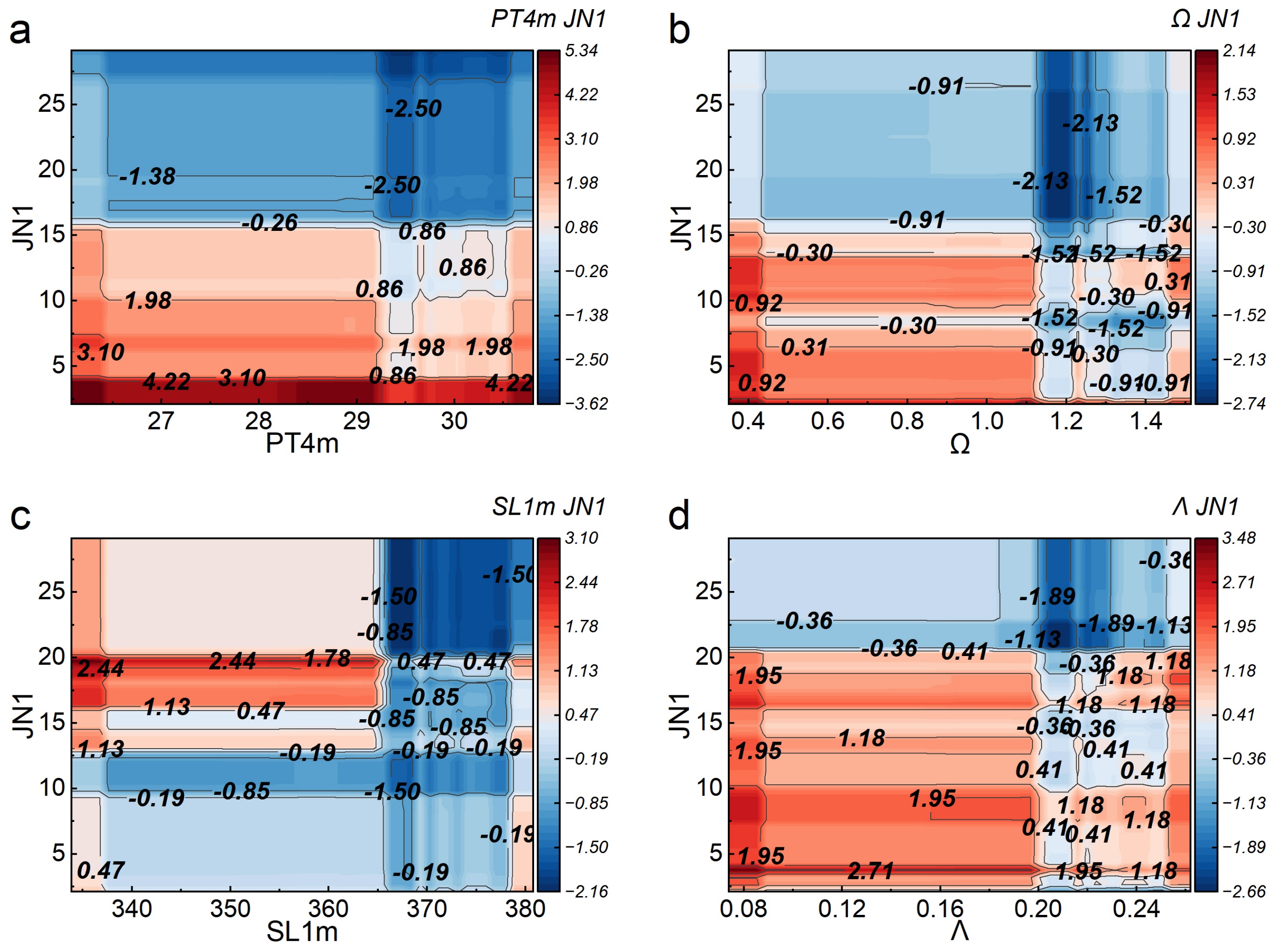


**Figure S7**. Two-dimensional PDP analysis for the four pairwise feature combinations. (a) PT4m-SL1m; (b) PT4m-Λ; (c) PT4m-JN1; (d) Ω-SL1m.


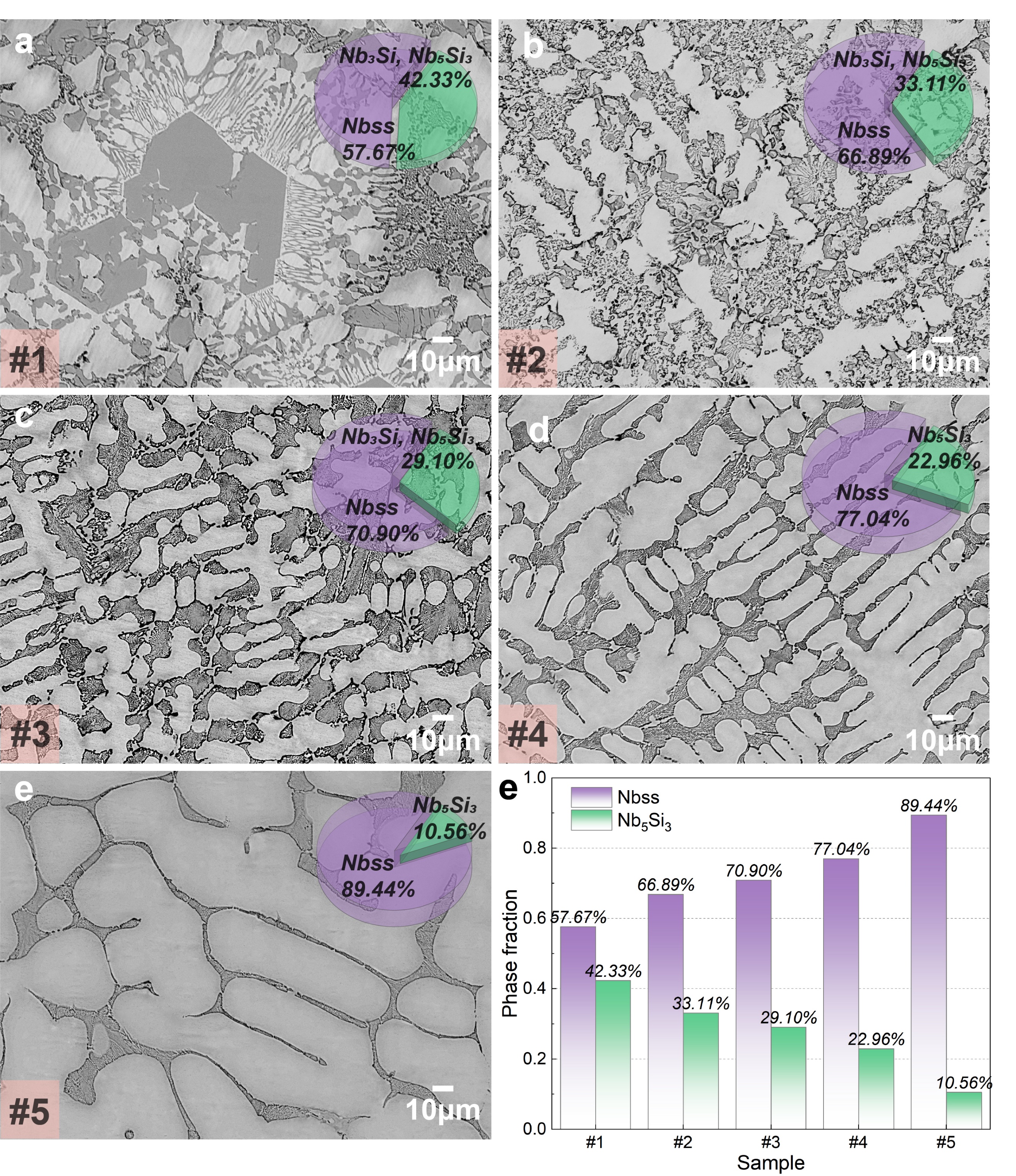


**Figure. S8**. SEM micrographs and phase fractions of alloys #1–#5. (a–e) Microstructures; (f) phase fraction summary.


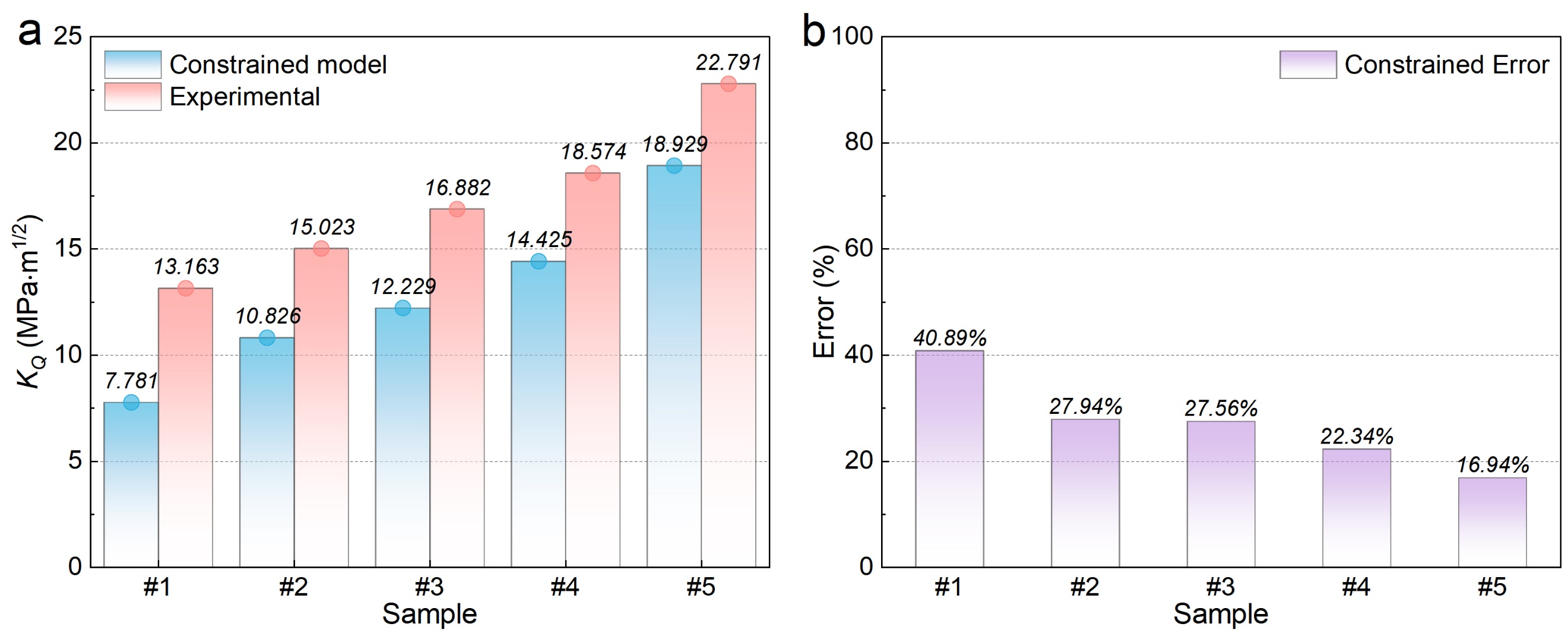


**Figure. S9**. Constrained model vs. experimental *K*_Q_ for alloys #1–#5. (a) Predicted vs. experimental values; (b) prediction errors.


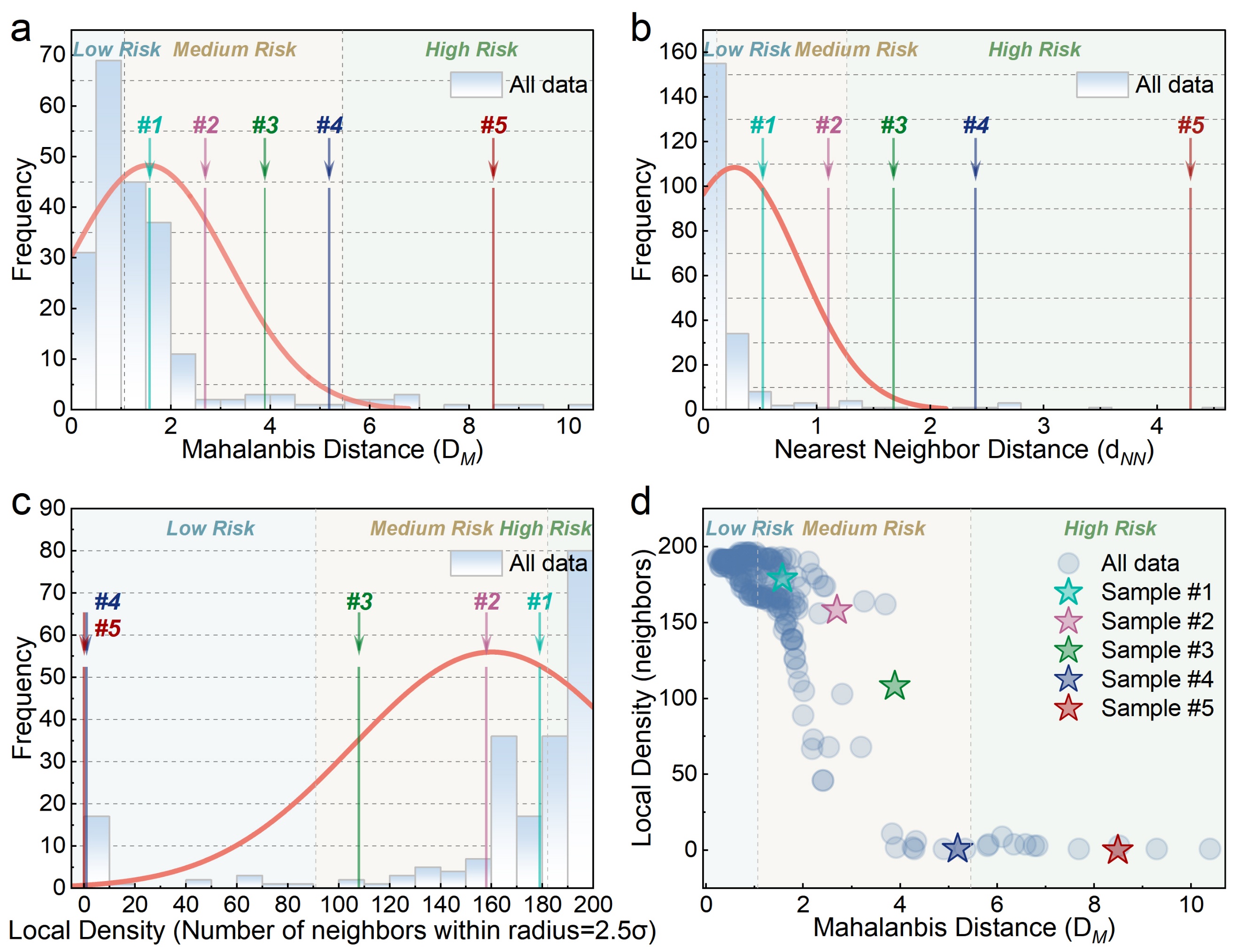


**Figure. S10**. Distance metric analysis for *K*_Q_ predictions. (a) Mahalanobis distance distribution with risk zones and optimized alloy positions, (b) nearest-neighbor distance distribution, (c) local density distribution using 2.5*σ* radius, and (d) scatter plot of Mahalanobis distance versus local density.


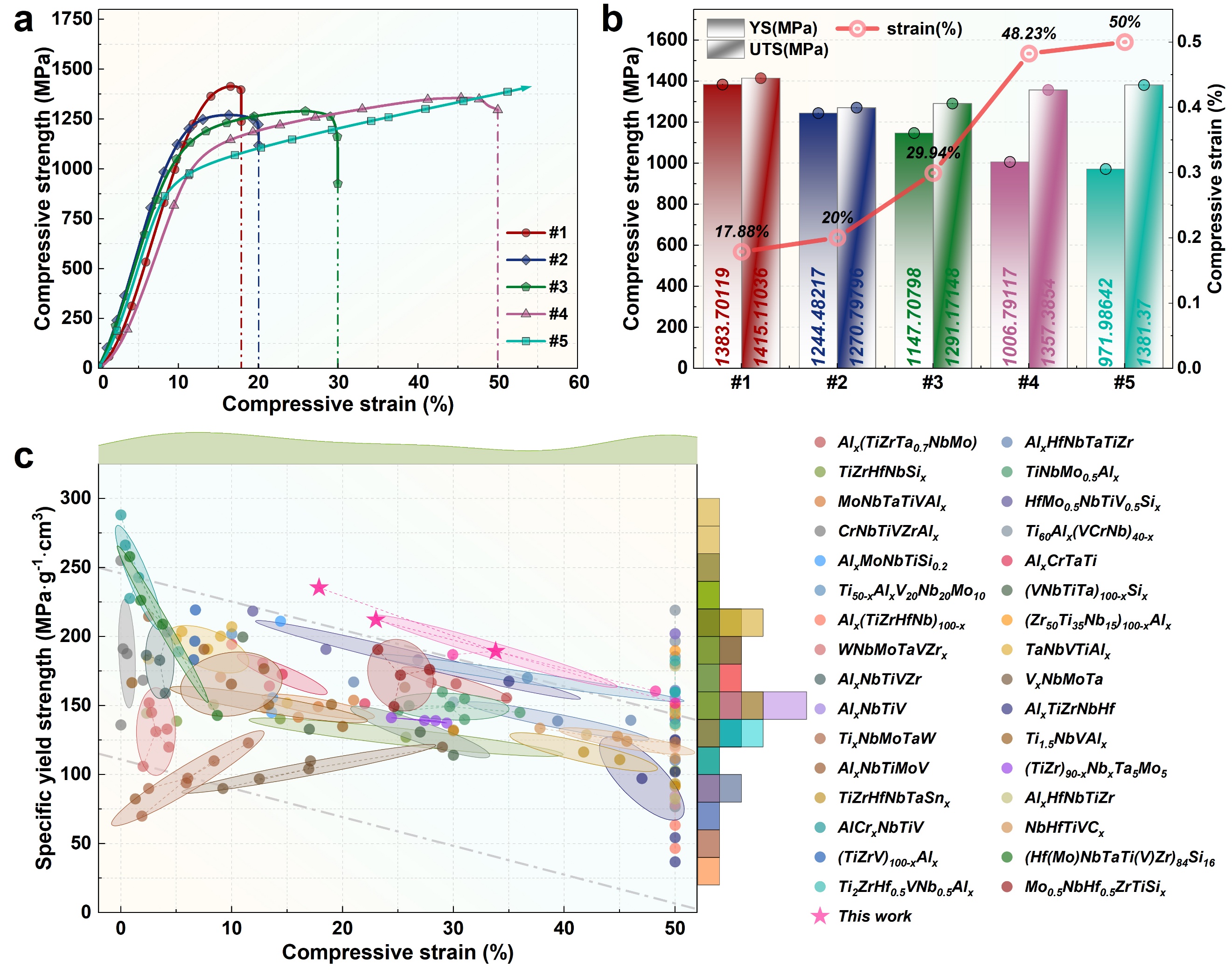


**Figure S11.** Compressive properties and comparison with other alloys for five validation alloy samples. (a) Room temperature compression test stress-strain curves; (b) Yield strength and ultimate compressive strength comparison graph and compressive strain curves; (c) Specific yield strength-compressive strain performance comparison with other alloys^[1-31]^.


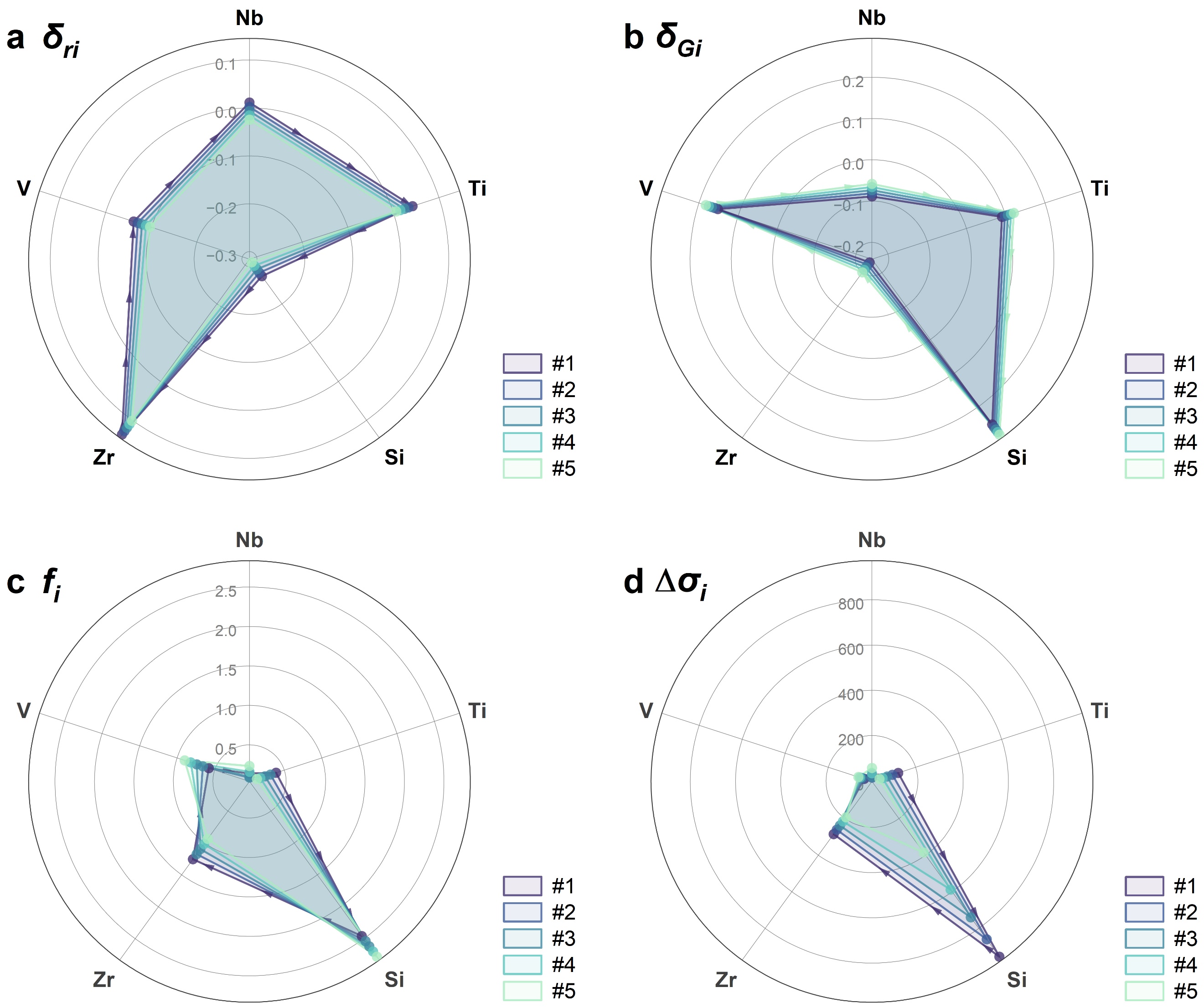


**Figure S12.** Solid solution strengthening analysis of samples. (a) Atomic radius difference parameter $\text{δ}_{\text{ri}}$; (b) Shear modulus difference parameter $\text{δ}_{\text{Gi}}$; (c) Volume fraction parameter $\text{f}_{\text{i}}$; (d) Strengthening increment parameter $\text{∆σ}_{\text{i}}$.


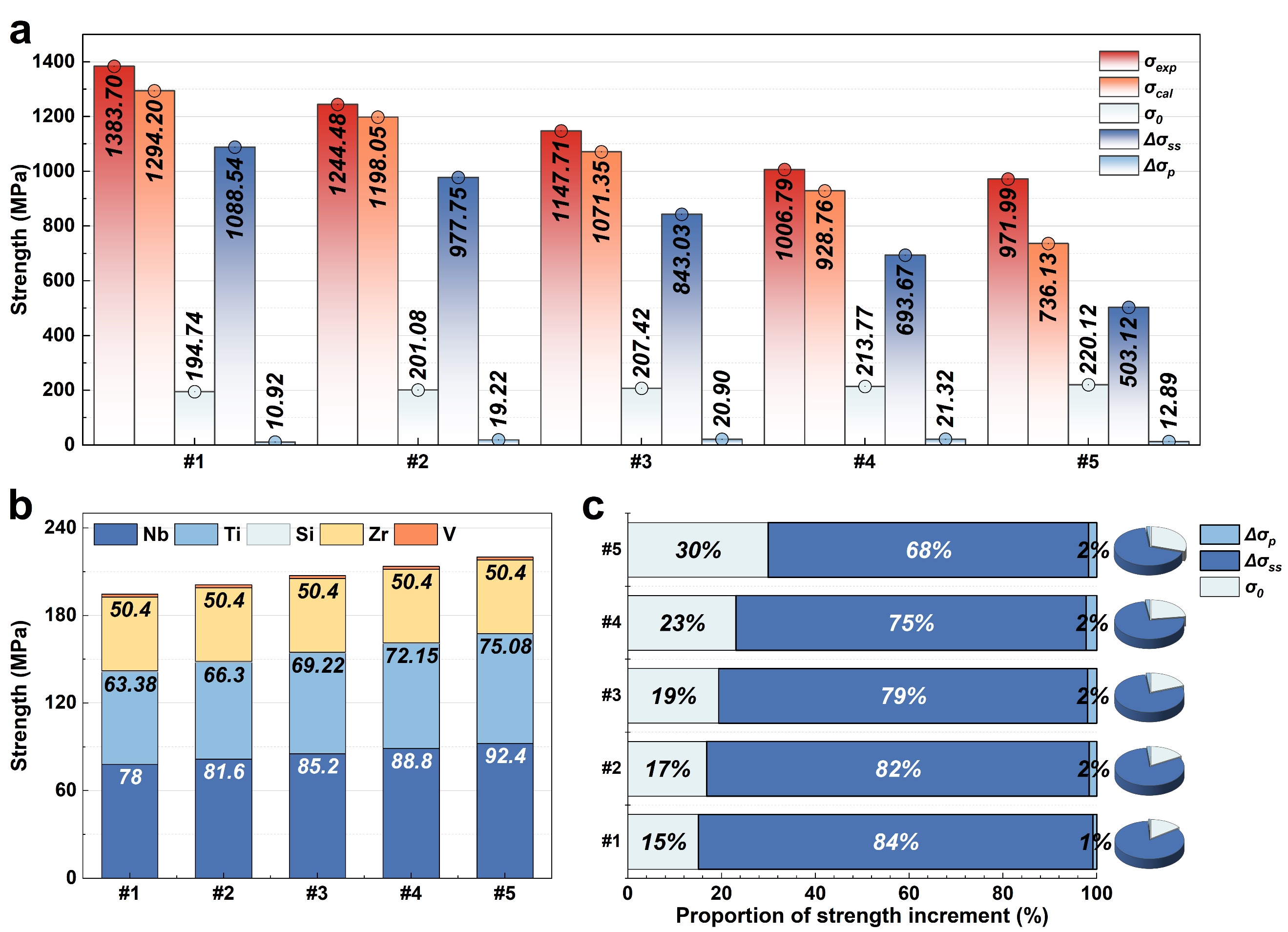


**Figure S13.** Strengthening mechanisms for validation samples. (a) Strengthening mechanism decomposition bar chart; (b) Specific contribution values of five alloying elements (Nb, Ti, Si, Zr, V) to intrinsic strength $\text{σ}_{\text{0}}$; (c) Relative proportions of three strengthening mechanisms.


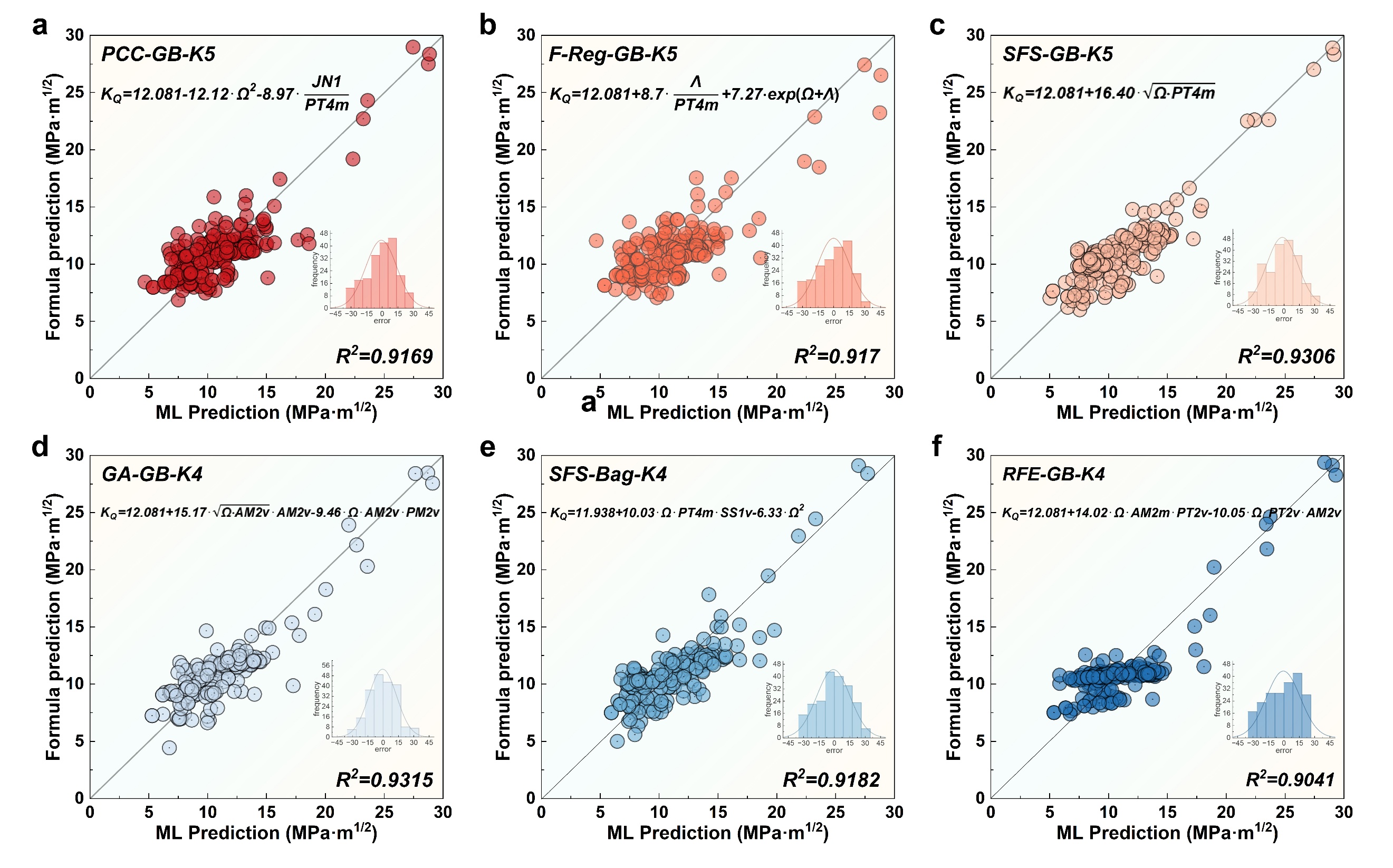


**Figure S14.** Formula fitting analysis for six models. (a) PCC-GB-K5; (b) F-Reg-GB-K5; (c) SFS-GB-K5; (d) GA-GB-K4; (e) SFS-Bag-K4; (f) RFE-GB-K4.


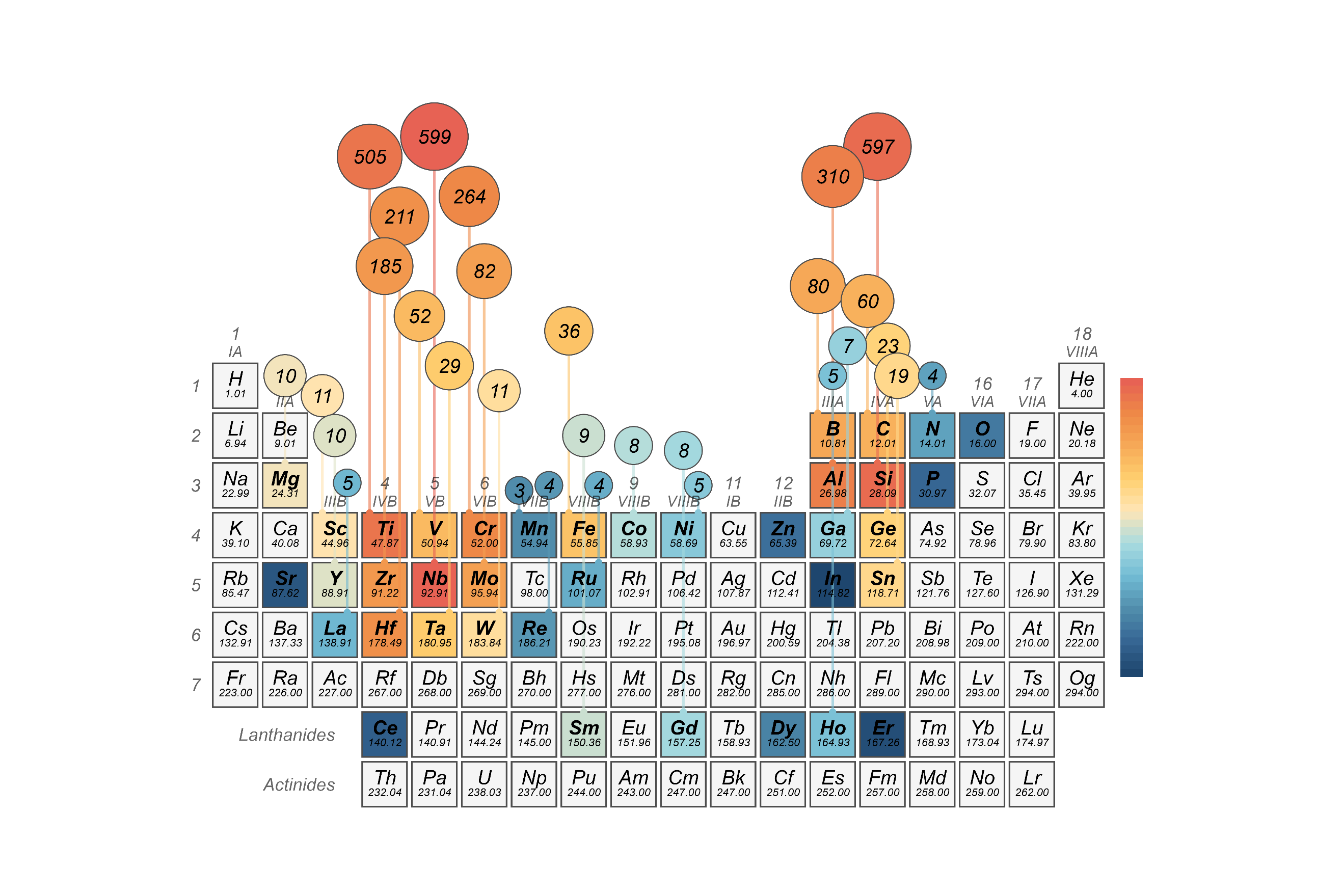


**Figure S15.** Element frequency distribution and dataset composition analysis for Nb-Si alloy database.


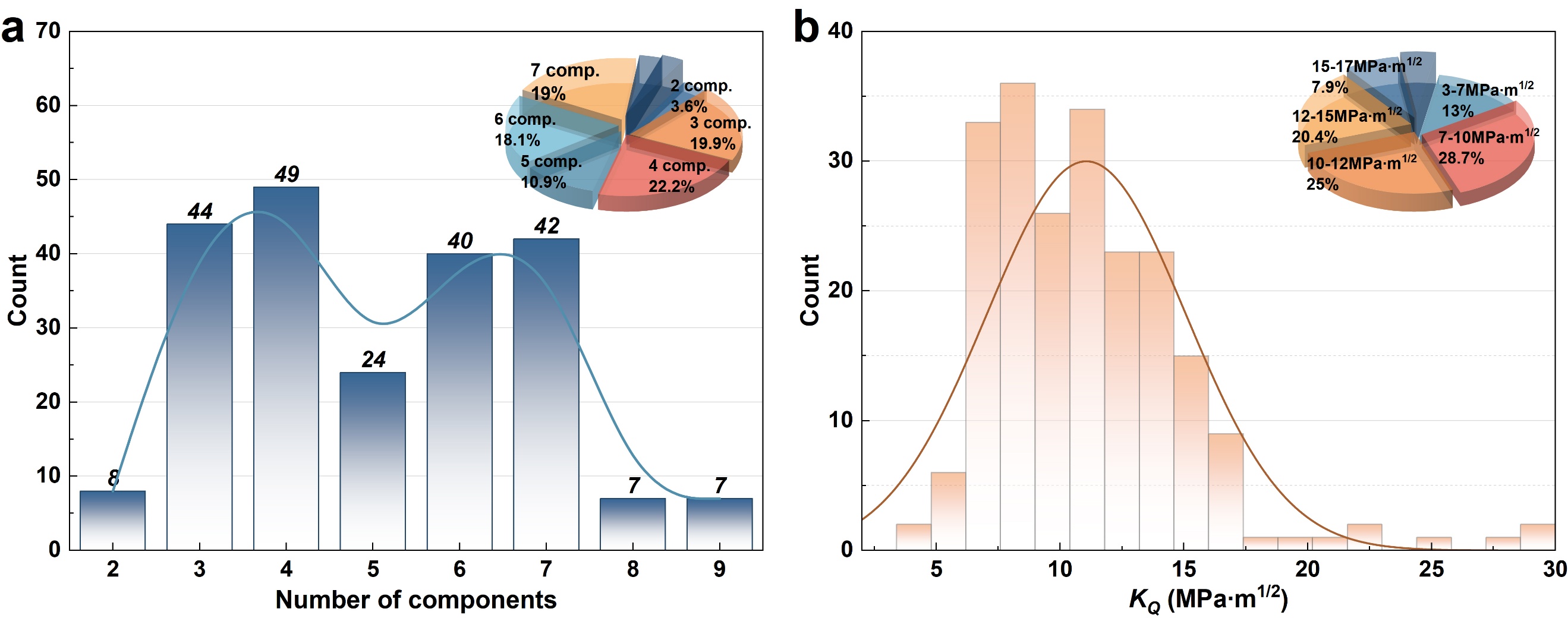


**Figure S16.** Database composition and target property analysis for Nb-Si alloys. (a) Alloy component number frequency statistics; (b) *K*_Q_ distribution characteristics.


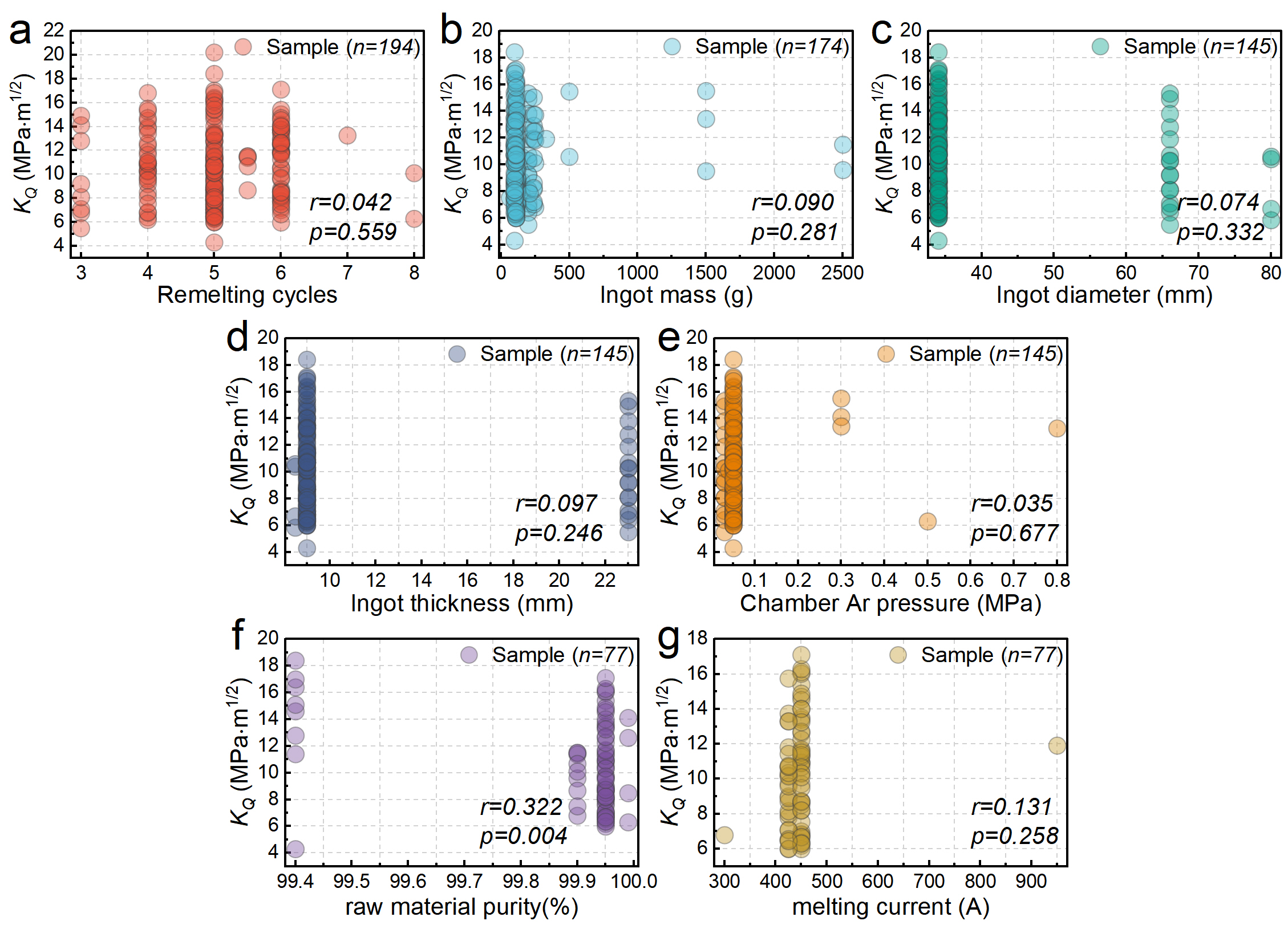


**Figure. S17.** Correlation between processing parameters and *K*_Q_. Scatter plots for (a) remelting cycles, (b) ingot mass, (c) ingot diameter, (d) ingot thickness, (e) argon pressure, (f) raw material purity, and (g) melting current with Pearson r and p-values.

**Tables**

**Table S1** Basic features for fracture toughness (*K*_Q_) prediction model.

| feature code | feature description | unit |
| --- | --- | --- |
| Nb | Nb (Niobium) | at.% |
| Si | Si (Silicon) | at.% |
| Ti | Ti (Titanium) | at.% |
| Hf | Hf (Hafnium) | at.% |
| Zr | Zr (Zirconium) | at.% |
| Al | Al (Aluminum) | at.% |
| Cr | Cr (Chromium) | at.% |
| Mo | Mo (Molybdenum) | at.% |
| V | V (Vanadium) | at.% |
| W | W (Tungsten) | at.% |
| Ta | Ta (Tantalum) | at.% |
| Sn | Sn (Tin) | at.% |
| VEC | Valence electron concentration | -- |
| ΔHmix | Mixing enthalpy | kJ/mol |
| ΔSmix | Mixing entropy | J/(mol·K) |
| ΔG | Gibbs free energy change | kJ/mol |
| Tm | Melting point | K |
| ΔTm | Melting point change | K |
| δ | δ (Atomic size difference parameter) | -- |
| Ω | Ω (Thermodynamic parameter Omega) | -- |
| $\text{χ}$ | Electronegativity parameter | -- |
| $\Delta\text{χ}$ | Electronegativity difference | -- |
| $\text{ρ}$ | Density parameter | g/cm³ |
| r | Atomic radius parameter | pm |
| am | Atomic mass parameter | -- |
| Δa | Lattice parameter difference | Å |
| Λ | Λ (Thermodynamic parameter Lambda) | -- |
| AE1 | Atomic number | -- |
| AW1 | Atomic weight | g/mol |
| PD1 | Density | g/cm³ |
| AE2 | Electronegativity (Pauling) | -- |
| AE4 | Electron affinity | kJ/mol |
| AE3 | Electrophilicity Index | eV |
| AR2 | Radii covalent | pm |
| AR4 | Covalent radius Pyykko(Single Bond) | pm |
| AR3 | Covalent radius Pyykko(Double Bond) | pm |
| AR5 | Covalent radius Pyykko(Triple Bond) | pm |
| AR1 | Radii atomic (coordination number 12) | pm |
| AR6 | Metallic radius | pm |
| AR7 | Metallic radius 12 Neighbors | pm |
| AM2 | Mass Magnetic Susceptibility | m³/kg |
| AM1 | Molar Magnetic Susceptibility | m³/mol |
| AM3 | Volume Magnetic Susceptibility | -- |
| AN1 | Neutron Cross Section | barns |
| AN2 | Neutron Mass Absorption | m²/kg |
| SS1 | Space Group Number | -- |
| AE5 | Glawe Number | -- |
| SL1 | Lattice Constants a | Å |
| SL2 | Lattice Constants b | Å |
| SL3 | Lattice Constants c | Å |
| PT1 | Temperature melting | K |
| PT2 | Temperature boiling | K |
| PT3 | Enthalpy vaporization | kJ/mol |
| PT4 | Enthalpy melting | kJ/mol |
| PT5 | Enthalpy atomization | kJ/mol |
| PT9 | Thermal expansion coefficient | K⁻¹ |
| PT7 | Specific heat capacity | J/(g·K) |
| PT6 | Molar heat capacity | J/(mol·K) |
| PD2 | Molar volume | cm³/mol |
| PT8 | Thermal conductivity | W/(m·K) |
| PE2 | Electrical resistivity | MS/m |
| PE1 | Resistivity | µΩ·m |
| PM2 | Young’s Modulus | Gpa |
| PM3 | Mohs hardness | - |
| PM1 | Speed of Sound | m/s |

**Table S2** Specific numerical values of elemental features.

|  | Nb | Si | Ti | Al | Cr | Hf | Zr | Mo | V | W | Ta | Sn |
| --- | --- | --- | --- | --- | --- | --- | --- | --- | --- | --- | --- | --- |
| AE1 | 41 | 14 | 22 | 13 | 24 | 72 | 40 | 42 | 23 | 74 | 73 | 50 |
| AW1 | 92.90637 | 28.085 | 47.867 | 26.98153 | 51.9961 | 178.49 | 91.224 | 95.95 | 50.9415 | 183.84 | 180.94788 | 118.71 |
| PD1 | 8.57 | 2.33 | 4.507 | 2.7 | 7.19 | 13.31 | 6.511 | 10.28 | 6.11 | 19.25 | 16.65 | 7.31 |
| AE2 | 1.6 | 1.9 | 1.54 | 1.61 | 1.66 | 1.3 | 1.33 | 2.16 | 1.63 | 2.36 | 1.5 | 1.96 |
| AE4 | 86.1 | 133.6 | 7.6 | 42.5 | 64.3 | 0 | 41.1 | 71.9 | 50.6 | 78.6 | 31 | 107.3 |
| AE3 | 1.261 | 1.683 | 0.884 | 0.927 | 1.132 | 0.858 | 1.004 | 1.211 | 1.062 | 1.336 | 1.072 | 1.434 |
| AR2 | 164 | 111 | 160 | 121 | 139 | 175 | 175 | 154 | 153 | 162 | 170 | 139 |
| AR4 | 147 | 116 | 136 | 126 | 122 | 152 | 154 | 138 | 134 | 137 | 146 | 140 |
| AR3 | 125 | 107 | 117 | 113 | 111 | 128 | 127 | 121 | 112 | 120 | 126 | 130 |
| AR5 | 116 | 102 | 108 | 111 | 103 | 122 | 121 | 113 | 106 | 115 | 119 | 132 |
| AR1 | 198 | 111 | 176 | 118 | 166 | 208 | 206 | 190 | 171 | 193 | 200 | 145 |
| AR6 | 134 | 117 | 132 | 125 | 119 | 144 | 145 | 130 | 122 | 130 | 134 | 142 |
| AR7 | 146 | 138 | 147 | 143 | 128 | 159 | 160 | 139 | 134 | 139 | 146 | 163 |
| AM2 | 27.6 | -1.6 | 40.1 | 7.8 | 44.5 | 5.3 | 16.8 | 11.7 | 62.8 | 4.59 | 10.7 | -3.1 |
| AM1 | 2.56 | -0.0449 | 1.919 | 0.21 | 2.314 | 0.946 | 1.53 | 1.122 | 3.199 | 0.844 | 1.936 | -0.368 |
| AM3 | 0.000237 | -0.00000373 | 0.0001807 | 0.0000211 | 0.0003177 | 0.0000705 | 0.000109 | 0.0001203 | 0.0003837 | 0.0000884 | 0.0001782 | -0.0000227 |
| AN1 | 1.15 | 0.166 | 6.1 | 0.233 | 3.1 | 104 | 0.184 | 2.6 | 5.06 | 18.4 | 20.5 | 0.62 |
| AN2 | 0.0004 | 0.0002 | 0.0044 | 0.03 | 0.0021 | 0.02 | 0.00066 | 0.0009 | 0.0033 | 0.0036 | 0.0041 | 0.0002 |
| SS1 | 229 | 227 | 194 | 225 | 229 | 194 | 194 | 229 | 229 | 229 | 229 | 141 |
| AE5 | 53 | 85 | 51 | 78 | 55 | 50 | 49 | 56 | 54 | 57 | 52 | 83 |
| SL1 | 330.04 | 543.09 | 295.08 | 404.95 | 291 | 319.64 | 323.2 | 314.7 | 303 | 316.52 | 330.13 | 905.4 |
| SL2 | 330.04 | 543.09 | 295.08 | 404.95 | 291 | 319.64 | 323.2 | 314.7 | 303 | 316.52 | 330.13 | 908.3 |
| SL3 | 330.04 | 543.09 | 468.55 | 404.95 | 291 | 505.11 | 514.7 | 314.7 | 303 | 316.52 | 330.13 | 1160.1 |
| PT1 | 2750 | 1687 | 1941 | 933.47 | 2180 | 2506 | 2128 | 2896 | 2183 | 3695 | 3290 | 505.08 |
| PT2 | 5017 | 3173 | 3560 | 2792 | 2944 | 4876 | 4682 | 4912 | 3650 | 5828 | 5731 | 2875 |
| PT3 | 680 | 383 | 422.6 | 284.1 | 342 | 575 | 567 | 590 | 460 | 824 | 758 | 296 |
| PT4 | 26.8 | 50.6 | 18.8 | 10.75 | 21 | 25.1 | 19.2 | 28 | 17.5 | 35 | 24.7 | 7.07 |
| PT5 | 733 | 450 | 473 | 330.9 | 397.48 | 618.4 | 610 | 658.98 | 515.5 | 851 | 782 | 301.2 |
| PT9 | 0.0000073 | 0.0000026 | 0.0000086 | 0.0000231 | 0.0000049 | 0.0000059 | 0.0000057 | 0.0000048 | 0.0000084 | 0.0000045 | 0.0000063 | 0.000022 |
| PT7 | 0.265 | 0.712 | 0.523 | 0.897 | 0.449 | 0.144 | 0.278 | 0.251 | 0.489 | 0.132 | 0.14 | 0.227 |
| PT6 | 24.6 | 19.789 | 25.06 | 24.2 | 23.35 | 25.73 | 25.36 | 24.06 | 24.89 | 24.27 | 25.36 | 27.112 |
| PD2 | 10.841 | 12.054 | 10.621 | 9.99 | 7.2317 | 13.4102 | 14.011 | 9.334 | 8.3374 | 9.5501 | 10.8677 | 16.239 |
| PT8 | 53.7 | 149 | 21.9 | 237 | 93.9 | 23 | 22.7 | 139 | 30.7 | 173 | 57.5 | 66.8 |
| PE2 | 6.7 | 0.001 | 2.5 | 38 | 7.9 | 3.3 | 2.4 | 20 | 5 | 20 | 7.7 | 9.1 |
| PE1 | 0.00000015 | 0.001 | 0.0000004 | 0.000000026 | 0.00000013 | 0.0000003 | 0.00000042 | 5E-08 | 0.0000002 | 5E-08 | 0.00000013 | 0.00000011 |
| PM2 | 105 | 47 | 116 | 70 | 279 | 78 | 68 | 329 | 128 | 411 | 186 | 50 |
| PM3 | 6 | 6.5 | 6 | 2.75 | 8.5 | 5.5 | 5 | 5.5 | 7 | 7.5 | 6.5 | 1.5 |
| PM1 | 3480 | 2200 | 4140 | 5100 | 5940 | 3010 | 3800 | 6190 | 4560 | 5174 | 3400 | 2500 |

Note: To extend from element-level features to alloy-level descriptors, alloy feature factors were calculated through weighted averages and weighted variances：

|  | $f_{mi}=\sum(f_{ij}\times\alpha_{j})/\sum\alpha_{j}$ | (S1) |
| --- | --- | --- |
|  | $f_{vi}=\sum\left[ \left( f_{ij}-f_{mi} \right)^{2}\times\alpha_{j} \right]/\sum\alpha_{j}$ | (S2) |

where $f_{ij}$ is the i-th feature value of element j; $\alpha_{j}$ is the atomic percentage of element j; $f_{mi}$ and $f_{vi}$ are the feature mean and variance factors of the alloy, respectively. The mean factor reflects the overall feature level of the alloy, while the variance factor characterizes the degree of heterogeneity among constituent elements. The resulting mean feature values are labeled with identifier “m”, and variance feature values are labeled with identifier “v”.

**Table S3** Physics-guided engineering features for fracture toughness (*K*_Q_) model.

| feature code | Feature Name | Calculation Formula | Physical Meaning |
| --- | --- | --- | --- |
| JT1 | Pugh modulus ratio | (VEC × 9.4 - 4.0) / (VEC × 4.2 + 25) | Pugh modulus ratio (B/G) based on VEC calculation |
| JT2 | Poisson ratio predicted | 0.33 - 0.015 × VEC | Predicted Poisson ratio based on VEC calculation |
| JT3 | Cauchy pressure indicator | -5.2 ×VEC + 45 | Cauchy pressure indicator based on VEC calculation |
| JT4 | Si supersaturation | max(0, Si - 0.08) | Si supersaturation |
| JT5 | Atomic size mismatch Nb-Si | Nb × Si × ((1.43 - 1.11) / 1.43)² | Nb-Si atomic size mismatch |
| JE1 | VEC Omega coupling | VEC ×Ω | VEC-Ω coupling |
| JE2 | VEC stabilization | VEC / (1 + abs(ΔHmix)) | VEC stabilization effect |
| JE3 | VEC deviation from ideal | abs(VEC - 4.5) | VEC deviation from ideal value |
| JE4 | Electron localization | AE2m / (AE2v + 1e-6) | Electron localization parameter |
| JH2 | Thermodynamic driving force | -ΔG / (R ×Tm) | Thermodynamic driving force |
| JH3 | Entropy stabilization | ΔSmix / R | Entropy stabilization effect |
| JH1 | Phase stability index | TH8 × exp(-abs(ΔHmix)/(R ×Tm)) | Phase stability index |
| JH4 | Thermal stability factor | TH5 / (abs(ΔTm) + 1) | Thermal stability factor |
| JN3 | Nb-Si ratio | CC1 / (Si + 1e-6) | Nb/Si ratio |
| JN2 | Eutectic distance | abs(Si - 0.16) | Eutectic distance |
| JN5 | Hypereutectic indicator | max(0, Si - 0.16) | Hypereutectic indicator |
| JN4 | Silicide volume fraction | Si × (1 - exp(-Si / 0.1)) | Silicide volume fraction prediction |
| JN1 | Solubility limit factor | Si / (exp(-1000 / PT1m) + 1e-6) | Solubility limit factor |
| JM3 | Toughness indicator | sqrt(PM2m × PD1m) / (AE2v + 1e-6) | Toughness indicator |
| JM4 | Crack resistance factor | PM2m / (PD1m × AR1v + 1e-6) | Crack resistance factor |
| JM5 | Plastic deformation capacity | JT1 × JT2 | Plastic deformation capacity indicator |
| JM1 | Stress concentration factor | δ× AR1v | Microscopic stress concentration factor |
| JM2 | Lattice distortion energy | δ² × PM2m | Lattice distortion energy |
| JD1 | Diffusion resistance | PT1m / (AR1m² + 1e-6) | Diffusion resistance |
| JD2 | Diffusion activation energy | 0.3 × PT1m × R + abs(ΔHmix) | Diffusion activation energy estimation |
| JA1 | Refractory strengthening | JA1a × PT1m / PD1m | Refractory elements strengthening effect |
| JA2 | Ti-Al synergy | Ti × Al × 2.0 | Ti-Al synergistic effect |
| JA3 | Cr oxidation resistance | Cr × AE2m / AR1m | Cr oxidation resistance effect |
| JS1 | Interface coherency | 1 / (1 + AR1v) | Interface coherency |
| JS2 | Lattice mismatch effect | SL1v × SL2v | Lattice mismatch effect |
| JS3 | Phase separation tendency | ΔHmix / (ΔSmix + 1e-6) | Phase separation tendency |
| JS4 | precipitation hardening potential | Si × PM2m × 0.1 | Precipitation hardening potential |
| JS5 | Elastic anisotropy | PM2m / (PM2m / (2 × (1 + JT2)) + 1e-6) | Elastic anisotropy parameter |
| JX1 | Enthalpy entropy competition | abs(ΔHmix) / (Tm ×ΔSmix + 1e-6) | Enthalpy-entropy competition effect |
| JX3 | VEC squared deviation | (VEC - 4.5)² | VEC squared deviation |

**Table S4** Feature selection methods.

| Category | Method | Abbreviation | Description |
| --- | --- | --- | --- |
| Traditional | Recursive Feature Elimination | RFE | Recursively eliminates features based on model performance |
| Traditional | F-regression | F-Reg | Uses F-test to select features based on linear relationship |
| Traditional | Pearson Correlation Coefficient | PCC | Selects features based on linear correlation with target |
| Traditional | Lasso Regularization | Lasso | Uses L1 regularization for automatic feature selection |
| Traditional | Random Forest | RF | Uses tree-based feature importance for selection |
| Traditional | SelectKBest | SKB | Selects k highest scoring features using univariate tests |
| Advanced | Sequential Forward Selection | SFS | Iteratively adds features that improve model performance |
| Advanced | Sequential Backward Selection | SBS | Iteratively removes features that least affect performance |
| Advanced | Genetic Algorithm | GA | Uses evolutionary algorithm for optimal feature subset |

**Table S5** Machine learning model.

| Category | Algorithm | Abbreviation | Type |
| --- | --- | --- | --- |
| Linear Models | Linear Regression | LR | Linear regression with ordinary least squares |
| Linear Models | Ridge Regression | Ridge | Linear regression with L2 regularization |
| Linear Models | Lasso Regression | Lasso | Linear regression with L1 regularization |
| Linear Models | Elastic Net | EN | Linear regression with L1 and L2 regularization |
| Linear Models | Bayesian Ridge | BR | Bayesian approach to ridge regression |
| Linear Models | Huber Regressor | HR | Robust linear regression less sensitive to outliers |
| Tree-based Models | Random Forest | RF | Ensemble of decision trees with bagging |
| Tree-based Models | Gradient Boosting | GB | Sequential ensemble of weak learners |
| Tree-based Models | Decision Tree | DT | Single decision tree regressor |
| Tree-based Models | XGBoost | XGB | Extreme gradient boosting implementation |
| Tree-based Models | CatBoost | CB | Gradient boosting optimized for categorical features |
| Tree-based Models | Histogram Gradient Boosting | HGB | Memory-efficient gradient boosting |
| Tree-based Models | AdaBoost | Ada | Adaptive boosting algorithm |
| Tree-based Models | Bagging | Bag | Bootstrap aggregating ensemble method |
| Distance-based Models | K-Nearest Neighbors | KNN | Prediction based on k nearest neighbors |
| Ensemble Methods | Voting Regressor | Vote | Combines predictions from multiple algorithms |

**Table S6** Key alloy factors affecting PCC-GB-K5 model.

| Feature | Description | Formula |
| --- | --- | --- |
| PT4m | Enthalpy melting | - |
| Ω | Thermodynamic parameter | $\frac{T_{m}\Delta S_{\mathrm{mix}}}{\vert\Delta H_{\mathrm{mix}}\vert}$ |
| SL1m | Lattice Constants a | - |
| Λ | Thermodynamic parameter | $\frac{\Delta S_{\mathrm{mix}}}{\delta^{2}}$ |
| JN1 | Solubility limit factor | $\frac{Si}{e^{-\frac{1000}{T_{m}}}+{10}^{-6}}$ |

**Table S7** Key alloy factors analysis results.

| model | PCC-GB-K5 | | | | |
| --- | --- | --- | --- | --- | --- |
| Key alloy factors | PT4m | Ω | SL1m | Λ | JN1 |
| Kni | -17.26 | 1.47 | -7.37 | 3.16 | -2.54 |
| Zni | 0.54 | 0.05 | 0.23 | 0.10 | 0.08 |

**Table S8** Compositions, feature values, and model predictions for validation alloys.

| Alloy | Nb | Si | Ti | Zr | V | PT4m | Ω | SL1m | Λ | JN1 | Predicted *K*_Q_ (MPa·m^1/2^) |
| --- | --- | --- | --- | --- | --- | --- | --- | --- | --- | --- | --- |
| #1 | 32.5 | 15 | 32.5 | 18 | 2 | 26.216 | 0.808 | 348.864 | 0.136 | 23.610 | 13.762 |
| #2 | 34 | 12 | 34 | 18 | 2 | 25.382 | 0.983 | 341.948 | 0.155 | 18.812 | 15.199 |
| #3 | 35.5 | 9 | 35.5 | 18 | 2 | 24.548 | 1.281 | 335.032 | 0.183 | 14.053 | 17.774 |
| #4 | 37 | 6 | 37 | 18 | 2 | 23.714 | 1.917 | 328.116 | 0.225 | 9.333 | 19.173 |
| #5 | 38.5 | 3 | 38.5 | 18 | 2 | 22.88 | 4.297 | 321.199 | 0.299 | 4.648 | 24.173 |

**Table S9** Comparison of experimental and predicted values.

| Alloy | Experiment |  |  | Prediction |  |  |  |
| --- | --- | --- | --- | --- | --- | --- | --- |
|  |  | PCC-GB-K5 | F-Reg-GB-K5 | SFS-GB-K5 | GA-GB-K4 | SFS-Bag-K4 | RFE-GB-K4 |
| #1 | 13.163 | 13.762 (+4.6%) | 13.847 (+5.2%) | 13.562 (+3.0%) | 13.928 (+5.8%) | 12.569 (-4.5%) | 13.528 (+2.8%) |
| #2 | 15.023 | 15.199 (+1.2%) | 15.199 (+1.2%) | 14.374 (-4.3%) | 14.883 (-0.9%) | 14.735 (-1.9%) | 14.087 (-6.2%) |
| #3 | 16.882 | 17.037 (+0.9%) | 15.917 (-5.7%) | 17.981 (+6.5%) | 15.836 (-6.2%) | 17.894 (+6.0%) | 15.789 (-6.5%) |
| #4 | 18.574 | 19.774 (+6.5%) | 19.687 (+6.0%) | 19.797 (+6.6%) | 18.917 (+1.8%) | 19.583 (+5.4%) | 19.536 (+5.2%) |
| #5 | 22.791 | 24.173 (+6.1%) | 23.984 (+5.2%) | 23.987 (+5.7%) | 23.996 (+5.3%) | 24.092 (+5.7%) | 24.281 (+6.5%) |

**Table S10** Constrained mixture model calculations and predictions.

| Alloy | Vb | Pc | $\left[ \frac{\mathrm{Kd}}{\mathrm{Kb}} \right]^{2}\mathrm{Pc}$ | K_IC_ pred | K_IC_ exp | Error (%) |
| --- | --- | --- | --- | --- | --- | --- |
| #1 | 0.4233 | 0.1414 | 16.09 | 7.781 | 13.163 | 40.89 |
| #2 | 0.3311 | 0.2671 | 30.40 | 10.826 | 15.023 | 27.94 |
| #3 | 0.2910 | 0.3348 | 38.09 | 12.229 | 16.882 | 27.56 |
| #4 | 0.2296 | 0.4517 | 51.41 | 14.425 | 18.574 | 22.34 |
| #5 | 0.1056 | 0.7299 | 83.07 | 18.929 | 22.791 | 16.94 |

Note: Kb = 3.0 MPa·m^1/2^, Kd = 32.0 MPa·m^1/2^, q' = 1.0.

**Table S11** Complete processing parameter database for all 216 samples with documented parameters.

| alloy | Purity | Atmosphere | Ar Pressure | Current | Remelts | Mass | Diameter | Thickness | Cooling |
| --- | --- | --- | --- | --- | --- | --- | --- | --- | --- |
| alloy1 | 99.90% | Ar | - | - | 5-6× | 2500g | - | - | Cu crucible |
| alloy2 | 99.90% | Ar | - | × | 6× | 2500g | - | - | Cu crucible |
| alloy3 | 99.90% | Ar | - | - | 6× | 2500g | - | - | Cu crucible |
| alloy4 | 99.90% | Ar | - | 200A-400A | 3× | 250g | - | - | Cu crucible |
| alloy5 | 99.95% | Ar | - | - | 6× | 150g | - | - | Cu crucible |
| alloy6 | 99.95% | Ar | - | - | 6× | 150g | - | - | Cu crucible |
| alloy7 | 99.95% | Ar | - | - | 6× | 150g | - | - | Cu crucible |
| alloy8 | 99.95% | Ar | - | - | 6× | 150g | - | - | Cu crucible |
| alloy9 | - | Ar | - | - | 4× | - | - | - | Cu crucible |
| alloy10 | - | Ar | - | - | 4× | - | - | - | Cu crucible |
| alloy11 | - | Ar | - | - | 4× | - | - | - | Cu crucible |
| alloy12 | - | Ar | - | - | 4× | - | - | - | Cu crucible |
| alloy13 | - | Ar | - | - | 4× | - | - | - | Cu crucible |
| alloy14 | 99.99% | Ar | - | - | ≥5× | 500g | - | - | Cu crucible |
| alloy15 | 99.99% | Ar | 0.3MPa | - | 3× | - | - | - | Cu crucible |
| alloy16 | - | Ar | 0.03MPa | - | 4× | 200g | 66mm | 23mm | Cu crucible |
| alloy17 | - | Ar | 0.03MPa | - | 4× | 200g | 66mm | 23mm | Cu crucible |
| alloy18 | - | Ar | 0.03MPa | - | 4× | 200g | 66mm | 23mm | Cu crucible |
| alloy19 | - | Ar | 0.03MPa | - | 4× | 200g | 66mm | 23mm | Cu crucible |
| alloy20 | - | Ar | 0.03MPa | - | ≥3× | 200g | 66mm | 23mm | Cu crucible |
| alloy21 | - | Ar | 0.03MPa | - | ≥3× | 200g | 66mm | 23mm | Cu crucible |
| alloy22 | - | Ar | 0.03MPa | - | ≥3× | 200g | 66mm | 23mm | Cu crucible |
| alloy23 | - | Ar | 0.03MPa | - | ≥3× | 200g | 66mm | 23mm | Cu crucible |
| alloy24 | - | Ar | 0.03MPa | - | ≥4× | 200g | 66mm | 23mm | Cu crucible |
| alloy25 | - | Ar | 0.03MPa | - | ≥4× | 200g | 66mm | 23mm | Cu crucible |
| alloy26 | - | Ar | 0.03MPa | - | ≥4× | 200g | 66mm | 23mm | Cu crucible |
| alloy27 | - | Ar | 0.03MPa | - | ≥4× | 200g | 66mm | 23mm | Cu crucible |
| alloy28 | - | Ar | 0.03MPa | - | ≥3× | 200g | 66mm | 23mm | Cu crucible |
| alloy29 | - | Ar | 0.03MPa | - | ≥3× | 200g | 66mm | 23mm | Cu crucible |
| alloy30 | - | Ar | 0.03MPa | - | 4× | 200g | 66mm | 23mm | Cu crucible |
| alloy31 | - | Ar | 0.03MPa | - | 4× | 200g | 66mm | 23mm | Cu crucible |
| alloy32 | - | Ar | - | - | 6× | 240g | - | - | Cu crucible |
| alloy33 | - | Ar | - | - | 6× | 240g | - | - | Cu crucible |
| alloy34 | - | Ar | - | - | 6× | 240g | - | - | Cu crucible |
| alloy35 | - | Ar | - | - | 6× | 240g | - | - | Cu crucible |
| alloy36 | - | Ar | - | - | 6× | 240g | - | - | Cu crucible |
| alloy37 | - | Ar | - | - | 6× | 250g | - | - | Cu crucible |
| alloy38 | - | Ar | - | - | 6× | 250g | - | - | Cu crucible |
| alloy39 | - | Ar | - | - | 6× | 250g | - | - | Cu crucible |
| alloy40 | - | Ar | - | - | 6× | 250g | - | - | Cu crucible |
| alloy41 | 99.95% | Ar | 0.05MPa | 300A-550A | 5× | 120g | 33-35mm | 8-10mm | Cu crucible |
| alloy42 | 99.95% | Ar | 0.05MPa | 300A-550A | 5× | 120g | 33-35mm | 8-10mm | Cu crucible |
| alloy43 | 99.95% | Ar | 0.05MPa | 300A-550A | 5× | 120g | 33-35mm | 8-10mm | Cu crucible |
| alloy44 | 99.95% | Ar | 0.05MPa | 300A-550A | 5× | 120g | 33-35mm | 8-10mm | Cu crucible |
| alloy45 | 99.95% | Ar | 0.05MPa | 300A-550A | 5× | 120g | 33-35mm | 8-10mm | Cu crucible |
| alloy46 | 99.95% | Ar | 0.05MPa | 300A-550A | 5× | 120g | 33-35mm | 8-10mm | Cu crucible |
| alloy47 | 99.95% | Ar | 0.05MPa | 300A-550A | 5× | 120g | 33-35mm | 8-10mm | Cu crucible |
| alloy48 | - | Ar | 0.05MPa | - | 6× | 100g | 33-35mm | 8-10mm | Cu crucible |
| alloy49 | - | Ar | 0.05MPa | - | 6× | 100g | 33-35mm | 8-10mm | Cu crucible |
| alloy50 | - | Ar | 0.05MPa | - | 6× | 100g | 33-35mm | 8-10mm | Cu crucible |
| alloy51 | - | Ar | 0.05MPa | - | 6× | 100g | 33-35mm | 8-10mm | Cu crucible |
| alloy52 | - | Ar | 0.05MPa | - | 6× | 100g | 33-35mm | 8-10mm | Cu crucible |
| alloy53 | - | Ar | 0.05MPa | - | 6× | 100g | 33-35mm | 8-10mm | Cu crucible |
| alloy54 | - | Ar | 0.05MPa | - | 6× | 100g | 33-35mm | 8-10mm | Cu crucible |
| alloy55 | - | Ar | 0.05MPa | - | ≥5× | 100g | 33-35mm | 8-10mm | Cu crucible |
| alloy56 | - | Ar | 0.05MPa | - | ≥5× | 100g | 33-35mm | 8-10mm | Cu crucible |
| alloy57 | - | Ar | 0.05MPa | - | ≥5× | 100g | 33-35mm | 8-10mm | Cu crucible |
| alloy58 | 99.40% | Ar | 0.05MPa | - | ≥5× | 100g | 33-35mm | 8-10mm | Cu crucible |
| alloy59 | 99.40% | Ar | 0.05MPa | - | ≥5× | 100g | 33-35mm | 8-10mm | Cu crucible |
| alloy60 | 99.40% | Ar | 0.05MPa | - | ≥5× | 100g | 33-35mm | 8-10mm | Cu crucible |
| alloy61 | 99.40% | Ar | 0.05MPa | - | ≥5× | 100g | 33-35mm | 8-10mm | Cu crucible |
| alloy62 | 99.40% | Ar | 0.05MPa | - | ≥5× | 100g | 33-35mm | 8-10mm | Cu crucible |
| alloy63 | 99.40% | Ar | 0.05MPa | - | ≥5× | 100g | 33-35mm | 8-10mm | Cu crucible |
| alloy64 | 99.40% | Ar | 0.05MPa | - | ≥5× | 100g | 33-35mm | 8-10mm | Cu crucible |
| alloy65 | 99.40% | Ar | 0.05MPa | - | ≥5× | 100g | 33-35mm | 8-10mm | Cu crucible |
| alloy66 | 99.40% | Ar | 0.05MPa | - | ≥5× | 100g | 33-35mm | 8-10mm | Cu crucible |
| alloy67 | 99.95% | Ar | 0.05MPa | 300A-600A | 5× | 110g | 33-35mm | 8-10mm | Cu crucible |
| alloy68 | 99.95% | Ar | 0.05MPa | 300A-600A | 5× | 110g | 33-35mm | 8-10mm | Cu crucible |
| alloy69 | 99.95% | Ar | 0.05MPa | 300A-600A | 5× | 110g | 33-35mm | 8-10mm | Cu crucible |
| alloy70 | 99.95% | Ar | 0.05MPa | 300A-600A | 5× | 110g | 33-35mm | 8-10mm | Cu crucible |
| alloy71 | 99.95% | Ar | 0.05MPa | 300A-600A | 5× | 110g | 33-35mm | 8-10mm | Cu crucible |
| alloy72 | 99.95% | Ar | 0.05MPa | 300A-600A | 5× | 110g | 33-35mm | 8-10mm | Cu crucible |
| alloy73 | 99.95% | Ar | 0.05MPa | 300A-600A | 5× | 110g | 33-35mm | 8-10mm | Cu crucible |
| alloy74 | 99.95% | Ar | 0.05MPa | 300A-600A | 5× | 110g | 33-35mm | 8-10mm | Cu crucible |
| alloy75 | 99.95% | Ar | 0.05MPa | 300A-600A | 5× | 110g | 33-35mm | 8-10mm | Cu crucible |
| alloy76 | 99.95% | Ar | 0.05MPa | 300A-600A | 5× | 110g | 33-35mm | 8-10mm | Cu crucible |
| alloy77 | 99.95% | Ar | 0.05MPa | 300A-600A | 5× | 110g | 33-35mm | 8-10mm | Cu crucible |
| alloy78 | 99.95% | Ar | 0.05MPa | 300A-600A | 5× | 110g | 33-35mm | 8-10mm | Cu crucible |
| alloy79 | 99.95% | Ar | 0.05MPa | 300A-600A | 5× | 110g | 33-35mm | 8-10mm | Cu crucible |
| alloy80 | - | Ar | - | - | 6× | 240g | - | - | Cu crucible |
| alloy81 | - | Ar | - | - | 6× | 240g | - | - | Cu crucible |
| alloy82 | - | Ar | - | - | 6× | 240g | - | - | Cu crucible |
| alloy83 | - | Ar | - | - | 6× | 240g | - | - | Cu crucible |
| alloy84 | - | Ar | - | - | 6× | 240g | - | - | Cu crucible |
| alloy85 | - | Ar | - | - | 6× | 240g | - | - | Cu crucible |
| alloy86 | - | Ar | - | - | 6× | 240g | - | - | Cu crucible |
| alloy87 | 99.95% | Ar | 0.05MPa | 300A-600A | 4× | 110g | 33-35mm | 8-10mm | Cu crucible |
| alloy88 | 99.95% | Ar | 0.05MPa | 300A-600A | 4× | 110g | 33-35mm | 8-10mm | Cu crucible |
| alloy89 | 99.95% | Ar | 0.05MPa | 300A-600A | 4× | 110g | 33-35mm | 8-10mm | Cu crucible |
| alloy90 | 99.95% | Ar | 0.05MPa | 300A-600A | 4× | 110g | 33-35mm | 8-10mm | Cu crucible |
| alloy91 | 99.95% | Ar | 0.05MPa | 300A-600A | 4× | 110g | 33-35mm | 8-10mm | Cu crucible |
| alloy92 | 99.95% | Ar | 0.05MPa | 300A-600A | 4× | 110g | 33-35mm | 8-10mm | Cu crucible |
| alloy93 | 99.95% | Ar | 0.05MPa | 300A-600A | 4× | 110g | 33-35mm | 8-10mm | Cu crucible |
| alloy94 | 99.95% | Ar | 0.05MPa | 300A-550A | 5× | 120g | 33-35mm | 8-10mm | Cu crucible |
| alloy95 | 99.95% | Ar | 0.05MPa | 300A-550A | 5× | 120g | 33-35mm | 8-10mm | Cu crucible |
| alloy96 | 99.95% | Ar | 0.05MPa | 300A-550A | 5× | 120g | 33-35mm | 8-10mm | Cu crucible |
| alloy97 | 99.95% | Ar | 0.05MPa | 300A-550A | 5× | 120g | 33-35mm | 8-10mm | Cu crucible |
| alloy98 | 99.95% | Ar | 0.05MPa | 300A-550A | 5× | 120g | 33-35mm | 8-10mm | Cu crucible |
| alloy99 | 99.95% | Ar | 0.05MPa | 300A-550A | 5× | 120g | 33-35mm | 8-10mm | Cu crucible |
| alloy100 | 99.95% | Ar | 0.05MPa | 300A-550A | 5× | 120g | 33-35mm | 8-10mm | Cu crucible |
| alloy101 | 99.95% | Ar | 0.05MPa | 300A-550A | 5× | 120g | 33-35mm | 8-10mm | Cu crucible |
| alloy102 | 99.95% | Ar | 0.05MPa | 300A-550A | 5× | 120g | 33-35mm | 8-10mm | Cu crucible |
| alloy103 | 99.95% | Ar | 0.05MPa | 300A-550A | 5× | 110g | 33-35mm | 8-10mm | Cu crucible |
| alloy104 | 99.95% | Ar | 0.05MPa | 300A-550A | 6× | 120g | 33-35mm | 8-10mm | Cu crucible |
| alloy105 | - | Ar | 10⁻⁴ mbar | - | 5× | - | - | - | Cu crucible |
| alloy106 | 99.95% | Ar | 0.05MPa | 300A-600A | 6× | 110g | 33-35mm | 8-10mm | Cu crucible |
| alloy107 | 99.95% | Ar | 0.05MPa | 300A-600A | 6× | 110g | 33-35mm | 8-10mm | Cu crucible |
| alloy108 | 99.95% | Ar | 0.05MPa | 300A-600A | 6× | 110g | 33-35mm | 8-10mm | Cu crucible |
| alloy109 | 99.95% | Ar | 0.05MPa | 300A-600A | 6× | 110g | 33-35mm | 8-10mm | Cu crucible |
| alloy110 | 99.95% | Ar | 0.05MPa | 300A-600A | 6× | 110g | 33-35mm | 8-10mm | Cu crucible |
| alloy111 | 99.95% | Ar | 0.05MPa | 300A-600A | 6× | 110g | 33-35mm | 8-10mm | Cu crucible |
| alloy112 | 99.95% | Ar | 0.05MPa | 300A-600A | 6× | 110g | 33-35mm | 8-10mm | Cu crucible |
| alloy113 | 99.95% | Ar | 0.05MPa | 300A-600A | 6× | 110g | 33-35mm | 8-10mm | Cu crucible |
| alloy114 | 99.95% | Ar | 0.05MPa | 300A-600A | 6× | 110g | 33-35mm | 8-10mm | Cu crucible |
| alloy115 | 99.95% | Ar | 0.05MPa | 300A-600A | 6× | 110g | 33-35mm | 8-10mm | Cu crucible |
| alloy116 | 99.95% | Ar | 0.05MPa | 300A-600A | 5× | 110g | 33-35mm | 8-10mm | Cu crucible |
| alloy117 | 99.95% | Ar | 0.05MPa | 300A-600A | 5× | 110g | 33-35mm | 8-10mm | Cu crucible |
| alloy118 | 99.95% | Ar | 0.05MPa | 300A-600A | 5× | 110g | 33-35mm | 8-10mm | Cu crucible |
| alloy119 | 99.95% | Ar | 0.05MPa | 300A-600A | 5× | 110g | 33-35mm | 8-10mm | Cu crucible |
| alloy120 | 99.90% | Ar | 0.05MPa | 300A-600A | 5-6× | 100g | 33-35mm | 8-10mm | Cu crucible |
| alloy121 | 99.90% | Ar | 0.05MPa | 300A-600A | 5-6× | 100g | 33-35mm | 8-10mm | Cu crucible |
| alloy122 | 99.90% | Ar | 0.05MPa | 300A-600A | 5-6× | 100g | 33-35mm | 8-10mm | Cu crucible |
| alloy123 | 99.90% | Ar | 0.05MPa | 300A-600A | 5-6× | 100g | 33-35mm | 8-10mm | Cu crucible |
| alloy124 | 99.95% | Ar | 0.05MPa | 300A-600A | 5× | 110g | 33-35mm | 8-10mm | Cu crucible |
| alloy125 | 99.95% | Ar | 0.05MPa | 300A-600A | 5× | 110g | 33-35mm | 8-10mm | Cu crucible |
| alloy126 | 99.95% | Ar | 0.05MPa | 300A-600A | 5× | 110g | 33-35mm | 8-10mm | Cu crucible |
| alloy127 | 99.95% | Ar | 0.05MPa | 300A-600A | 5× | 110g | 33-35mm | 8-10mm | Cu crucible |
| alloy128 | 99.95% | Ar | 0.05MPa | 300A-600A | 5× | 110g | 33-35mm | 8-10mm | Cu crucible |
| alloy129 | - | Ar | - | - | 5× | - | - | - | Cu crucible |
| alloy130 | - | Ar | - | - | 5× | - | - | - | Cu crucible |
| alloy131 | - | Ar | - | - | 5× | - | - | - | Cu crucible |
| alloy132 | - | Ar | - | - | 5× | - | - | - | Cu crucible |
| alloy133 | - | Ar | - | - | 5× | 210g | - | - | Cu crucible |
| alloy134 | - | Ar | - | - | 4× | - | - | - | Cu crucible |
| alloy135 | - | Ar | - | - | 4× | - | - | - | Cu crucible |
| alloy136 | - | Ar | - | - | 4× | - | - | - | Cu crucible |
| alloy137 | - | Ar | - | - | 4× | - | - | - | Cu crucible |
| alloy138 | - | Ar | - | - | 5× | - | - | - | Cu crucible |
| alloy139 | - | Ar | - | - | 5× | - | - | - | Cu crucible |
| alloy140 | - | Ar | - | - | 5× | - | - | - | Cu crucible |
| alloy141 | - | - | - | - | - | - | - | - | Cu crucible |
| alloy142 | - | - | - | - | - | - | - | - | Cu crucible |
| alloy143 | - | - | - | - | - | - | - | - | Cu crucible |
| alloy144 | - | - | - | - | - | - | - | - | Cu crucible |
| alloy145 | - | - | - | - | - | - | - | - | Cu crucible |
| alloy146 | - | Ar | - | - | ≥5× | 500g | - | - | Cu crucible |
| alloy147 | - | Ar | - | - | ≥5× | 500g | - | - | Cu crucible |
| alloy148 | 99.90% | Ar | - | - | 6× | 65g | - | - | Cu crucible |
| alloy149 | - | Ar | - | - | - | - | 80mm | 8mm-9mm | Cu crucible |
| alloy150 | - | Ar | - | - | - | - | 80mm | 8mm-9mm | Cu crucible |
| alloy151 | - | Ar | - | - | - | - | 80mm | 8mm-9mm | Cu crucible |
| alloy152 | - | Ar | - | - | - | - | 80mm | 8mm-9mm | Cu crucible |
| alloy153 | 99.95% | Ar | 0.05MPa | 300A-600A | 5× | 110g | 33-35mm | 8-10mm | Cu crucible |
| alloy154 | 99.95% | Ar | 0.05MPa | 300A-600A | 5× | 110g | 33-35mm | 8-10mm | Cu crucible |
| alloy155 | 99.95% | Ar | 0.05MPa | 300A-600A | 5× | 110g | 33-35mm | 8-10mm | Cu crucible |
| alloy156 | 99.95% | Ar | 0.05MPa | 300A-600A | 5× | 110g | 33-35mm | 8-10mm | Cu crucible |
| alloy157 | 99.95% | Ar | 0.05MPa | 300A-600A | 5× | 110g | 33-35mm | 8-10mm | Cu crucible |
| alloy158 | 99.95% | Ar | 0.05MPa | 300A-600A | 5× | 110g | 33-35mm | 8-10mm | Cu crucible |
| alloy159 | - | Ar | - | - | - | - | - | - | Cu crucible |
| alloy160 | 99.95% | Ar | 0.03MPa | 300A-600A | 5× | 110g | 33-35mm | 8-10mm | Cu crucible |
| alloy161 | 99.90% | Ar | 0.04MPa | 300A-600A | 8× | 100g | 33-35mm | 8-10mm | Cu crucible |
| alloy162 | 99.95% | Ar | <0.8MPa | 300A-600A | 6-8× | 110g | 33-35mm | 8-10mm | Cu crucible |
| alloy163 | 99.99% | Ar | 0.5MPa | 300A-600A | 8× | 110g | 33-35mm | 8-10mm | Cu crucible |
| alloy164 | 99.99% | Ar | - | - | 6× | 100g | - | - | Cu crucible |
| alloy165 | 99.99% | Ar | - | - | 6× | 100g | - | - | Cu crucible |
| alloy166 | - | Ar | - | - | ≥4× | - | - | - | Cu crucible |
| alloy167 | - | Ar | - | - | ≥4× | - | - | - | Cu crucible |
| alloy168 | - | Ar | - | - | ≥4× | - | - | - | Cu crucible |
| alloy169 | - | Ar | - | - | ≥4× | - | - | - | Cu crucible |
| alloy170 | - | Ar | - | - | ≥4× | - | - | - | Cu crucible |
| alloy171 | - | Ar | - | - | ≥4× | - | - | - | Cu crucible |
| alloy172 | - | Ar | 0.05MPa | 300A-600A | 6× | 120g | 33-35mm | 8-10mm | Cu crucible |
| alloy173 | - | Ar | 0.05MPa | - | 5× | 100g | 33-35mm | 8-10mm | Cu crucible |
| alloy174 | - | Ar | 0.05MPa | - | 5× | 100g | 33-35mm | 8-10mm | Cu crucible |
| alloy175 | - | Ar | 0.05MPa | - | 5× | 100g | 33-35mm | 8-10mm | Cu crucible |
| alloy176 | - | Ar | 0.05MPa | - | 5× | 100g | 33-35mm | 8-10mm | Cu crucible |
| alloy177 | - | Ar | 0.05MPa | - | 5× | 100g | 33-35mm | 8-10mm | Cu crucible |
| alloy178 | - | Ar | 0.05MPa | - | 5× | 100g | 33-35mm | 8-10mm | Cu crucible |
| alloy179 | - | Ar | 0.05MPa | - | 5× | 100g | 33-35mm | 8-10mm | Cu crucible |
| alloy180 | - | Ar | 0.05MPa | - | 5× | 100g | 33-35mm | 8-10mm | Cu crucible |
| alloy181 | - | Ar | 0.05MPa | - | 5× | 100g | 33-35mm | 8-10mm | Cu crucible |
| alloy182 | - | Ar | 0.05MPa | - | 5× | 100g | 33-35mm | 8-10mm | Cu crucible |
| alloy183 | - | Ar | 0.05MPa | - | 5× | 100g | 33-35mm | 8-10mm | Cu crucible |
| alloy184 | - | Ar | 0.05MPa | - | 5× | 100g | 33-35mm | 8-10mm | Cu crucible |
| alloy185 | - | Ar | 0.05MPa | - | 5× | 100g | 33-35mm | 8-10mm | Cu crucible |
| alloy186 | - | Ar | 0.05MPa | - | 5× | 100g | 33-35mm | 8-10mm | Cu crucible |
| alloy187 | - | Ar | 0.2-0.4MPa | - | ≥4× | 1500g | - | - | Cu crucible |
| alloy188 | - | Ar | 0.2-0.4MPa | - | ≥4× | 1500g | - | - | Cu crucible |
| alloy189 | - | Ar | 0.05MPa | - | 5× | 100g | 33-35mm | 8-10mm | Cu crucible |
| alloy190 | - | Ar | 0.05MPa | - | 5× | 100g | 33-35mm | 8-10mm | Cu crucible |
| alloy191 | - | Ar | 0.05MPa | - | 5× | 100g | 33-35mm | 8-10mm | Cu crucible |
| alloy192 | - | Ar | 0.05MPa | - | 5× | 100g | 33-35mm | 8-10mm | Cu crucible |
| alloy193 | - | Ar | 0.05MPa | - | 5× | 100g | 33-35mm | 8-10mm | Cu crucible |
| alloy194 | - | Ar | 0.05MPa | - | 5× | 100g | 33-35mm | 8-10mm | Cu crucible |
| alloy195 | - | Ar | 0.05MPa | - | 5× | 100g | 33-35mm | 8-10mm | Cu crucible |
| alloy196 | - | Ar | 0.05MPa | - | 5× | 100g | 33-35mm | 8-10mm | Cu crucible |
| alloy197 | - | Ar | 0.05MPa | - | 5× | 100g | 33-35mm | 8-10mm | Cu crucible |
| alloy198 | - | Ar | 0.05MPa | - | 5× | 100g | 33-35mm | 8-10mm | Cu crucible |
| alloy199 | - | Ar | 0.05MPa | - | 5× | 100g | 33-35mm | 8-10mm | Cu crucible |
| alloy200 | - | Ar | 0.05MPa | - | 5× | 100g | 33-35mm | 8-10mm | Cu crucible |
| alloy201 | - | Ar | 0.05MPa | - | 5× | 100g | 33-35mm | 8-10mm | Cu crucible |
| alloy202 | - | Ar | 0.05MPa | - | 5× | 100g | 33-35mm | 8-10mm | Cu crucible |
| alloy203 | - | Ar | 0.05MPa | - | 5× | 100g | 33-35mm | 8-10mm | Cu crucible |
| alloy204 | - | Ar | 0.05MPa | - | 5× | 100g | 33-35mm | 8-10mm | Cu crucible |
| alloy205 | - | Ar | 0.05MPa | - | 5× | 100g | 33-35mm | 8-10mm | Cu crucible |
| alloy206 | - | Ar | 0.05MPa | - | 5× | 100g | 33-35mm | 8-10mm | Cu crucible |
| alloy207 | - | Ar | 0.05MPa | 300A-550A | 5× | 110g | 33-35mm | 8-10mm | Cu crucible |
| alloy208 | - | Ar | 0.05MPa | 300A-550A | 5× | 110g | 33-35mm | 8-10mm | Cu crucible |
| alloy209 | - | Ar | 0.05MPa | 300A-550A | 5× | 110g | 33-35mm | 8-10mm | Cu crucible |
| alloy210 | - | Ar | 0.05MPa | 300A-550A | 5× | 110g | 33-35mm | 8-10mm | Cu crucible |
| alloy211 | - | Ar | 0.05MPa | 300A-550A | 5× | 110g | 33-35mm | 8-10mm | Cu crucible |
| alloy212 | - | Ar | 0.05MPa | 300A-550A | 5× | 110g | 33-35mm | 8-10mm | Cu crucible |
| alloy213 | - | Ar | 0.05MPa | 300A-550A | 5× | 110g | 33-35mm | 8-10mm | Cu crucible |
| alloy214 | - | Ar | 0.05MPa | 300A-550A | 5× | 110g | 33-35mm | 8-10mm | Cu crucible |
| alloy215 | - | Ar | 0.05MPa | 300A-550A | 5× | 110g | 33-35mm | 8-10mm | Cu crucible |
| alloy216 | - | Ar | 0.05MPa | - | 4× | 1500g | 10cm | - | Cu crucible |

Note: "-" indicates parameter not reported in original publication.

**Table S12** Summary statistics for processing parameters across the dataset.

| Parameter | n | Documentation Rate | Range | Mean ± SD | Median | Most Common Value(s) |
| --- | --- | --- | --- | --- | --- | --- |
| Remelting cycles | 206 | 95.4% | 3-8times | 5.0 ± 0.8 | 5 | 5 (53.9%) |
| Ingot mass | 182 | 84.3% | 65-2500 g | 198 ± 352g | 110g | 100-110 g (63.7%) |
| Ingot diameter | 152 | 70.4% | 33-100 mm | - | - | 33-35 mm (86.2%) |
| Ingot thickness | 152 | 70.4% | 8-23 mm | - | - | 8-10 mm (89.5%) |
| Chamber Ar pressure | 152 | 70.4% | 0.03-0.8 MPa | - | 0.05 MPa | 0.05 MPa (84.2%) |
| Protective atmosphere | 198 | 97.5% | Ar gas | - | - | Ar (100%) |
| Cooling method | 203 | 100% | Water-cooled Cu | - | - | Cu crucible (100%) |
| Raw material purity | 93 | 43.1% | 99.4-99.99% | - | 99.95% | 99.95% (74.2%) |
| Melting current | 82 | 38.0% | 200-1000 A | - | - | 300-600 A (98.8%) |

Notes: "-" indicates not applicable or not calculated for categorical/non-normal distributions.

**Table S13** Variance decomposition for *K*_Q_ dataset.

| Source of Variation | Variance *σ* (MPa·m^1/2^) | Percentage (%) | Combined Magnitude | Estimation Method |
| --- | --- | --- | --- | --- |
| Compositional factors | 3.98 | ~98% | - | Test R² = 0.96 |
| Processing variations | 0.4-0.5 | ~1-2% | - | Residual analysis |
| Measurement uncertainties | ~0.2 | ~0.2% | - | Remainder from metrics |
| Processing + Measurement | 0.45-0.54 | ~1-2% | 0.45-0.54 | $\text{σ}_{\text{unexplained}}$ |
| Total (observed) | 4.02 | 100% | - | Standard deviation |

**Supplementary Methods**

**Section S1 Residual analysis and prediction uncertainty quantification of the *K*_Q_ model**

**S1.1 Residual analysis**

Residual analysis was performed on the PCC-GB-K5 model to confirm the absence of systematic bias across the prediction range. Figure S2a shows residuals plotted against predicted *K*_Q_ values for both training and test sets. All data points fall within the ±2*σ* envelope with no monotonic trend or curvature across the full prediction range, ruling out heteroscedasticity and confirming that prediction error does not vary systematically with the magnitude of predicted *K*_Q_. The mean residuals are 0.0525 MPa·m^1/2^ (training) and −0.2431 MPa·m^1/2^ (test), indicating no meaningful systematic offset between the two sets.

Figure S2b shows the residual histograms for training and test sets separately. Figure S2c shows the Q-Q plot for the training set, where residuals align closely with the theoretical normal reference line, supported by a Shapiro-Wilk test yielding p = 0.8149, well above the 0.05 threshold, confirming that normality cannot be rejected for the training set. Figure S2d shows the Q-Q plot for the test set where residuals likewise align closely with the theoretical normal reference line, with a Shapiro-Wilk test yielding p = 0.8335, confirming normality for the test set. Figure S2e shows the autocorrelation function with all ACF values at non-zero lags remaining within the 95% confidence interval, confirming no serial correlation among prediction errors. Figure S2f shows the residual trend plot with the 3-point moving average remaining close to zero throughout, with fluctuations contained within the ±1*σ* bands. Taken together, these analyses confirm that prediction errors carry no structured information and that the model satisfies standard assumptions for reliable regression without systematic bias.

**S1.2 Prediction uncertainty quantification**

Two independent uncertainty quantification methods were applied to provide prediction intervals for the *K*_Q_ model: bootstrapped ensembles and quantile regression.

For the bootstrapped ensemble approach, N = 500 independent models were trained on resampled training sets and their predictions aggregated to obtain uncertainty estimates. Figure S3a shows training set predictions with ±1*σ* error bars and mean *σ* = 1.23 MPa·m^1/2^, confirming that prediction uncertainty remains consistent across the full *K*_Q_ range. Figure S3b presents test set results with ±1*σ* error bars and mean *σ* = 1.20 MPa·m^1/2^, demonstrating that uncertainty estimates appropriately capture prediction variability. Figure S3c shows the training set prediction uncertainty distribution with mean = 1.23 MPa·m^1/2^, standard deviation = 0.89 MPa·m^1/2^, minimum = 0.33 MPa·m^1/2^, and maximum = 5.93 MPa·m^1/2^. Figure S3d presents the test set uncertainty distribution with mean = 1.20 MPa·m^1/2^, standard deviation = 0.77 MPa·m^1/2^, minimum = 0.44 MPa·m^1/2^, and maximum = 3.57 MPa·m^1/2^. The similar mean uncertainty values between training and test sets confirm reasonable generalization. Figure S3e shows predicted *K*_Q_ values with 95% confidence intervals plotted against actual values across all test samples sorted by actual value, where the predicted values closely track actual measurements throughout the full *K*_Q_ range.

Quantile regression was applied as an independent alternative approach for prediction interval estimation. Figure S4a shows training set predictions with ±1*σ* error bars and mean *σ* = 2.16 MPa·m^1/2^. Figure S4b presents test set results with ±1*σ* error bars and mean *σ* = 2.20 MPa·m^1/2^. Figure S4c shows the training set uncertainty distribution with mean = 2.16 MPa·m^1/2^, standard deviation = 0.35 MPa·m^1/2^, minimum = 1.75 MPa·m^1/2^, and maximum = 4.22 MPa·m^1/2^. Figure S4d presents the test set uncertainty distribution with mean = 2.20 MPa·m^1/2^, standard deviation = 0.49 MPa·m^1/2^, minimum = 1.91 MPa·m^1/2^, and maximum = 4.22 MPa·m^1/2^. The quantile regression yields slightly wider prediction intervals than the bootstrapped ensemble, as it directly models the conditional distribution of *K*_Q_ rather than aggregating point predictions. Figure S4e shows that the 95% confidence intervals from quantile regression successfully bracket all actual *K*_Q_ measurements across the full test set. The two methods together provide well-calibrated prediction intervals, confirming that *K*_Q_ predictions carry a quantifiable uncertainty of approximately 1.2-2.2 MPa·m^1/2^ under standard conditions.

**Section S2. Element Importance Ranking Method Based on SHAP Analysis**

Through machine learning feature screening, key alloy factors affecting *K*_Q_ of Nb-Si alloys and their corresponding key feature quantities were identified. Table S6 shows the key factors identified after three-step progressive feature screening.

To quantitatively evaluate the influence of each key alloy factor on target properties, SHAP analysis was employed to establish the mapping relationship from microscopic features to macroscopic properties. This systematic approach enables quantitative element ranking based on their weighted contributions to fracture toughness performance.

Based on the feature screening results, {PT4m, Ω, SL1m, Λ, JN1} were defined as the key alloy factors influencing *K*_Q_. SHAP analysis was employed to interpret the black-box model for *K*_Q_ properties, with results presented in Figure S5a. Each data point represents an individual alloy sample, with the color indicating the magnitude of the corresponding feature value. By performing linear regression between normalized feature values and SHAP values, the influence coefficients kni for key features were obtained (Table S7). Normalized weight coefficients Zni were calculated based on |kni| to quantify the relative importance of each feature. The specific process is detailed below.

**S2.1 Determination of influence coefficients for key feature quantities**

First, all key alloy factors were normalized:

|  | $r_{Ej}^{norm}=\frac{r_{Ej}-r_{j}^{min}}{r_{j}^{max}-r_{j}^{min}}$ | (S3) |
| --- | --- | --- |

where $r_{Ej}$ is the j-th sample value of key alloy factor E; $r_{j}^{min}$ and $r_{j}^{max}$ are the minimum and maximum values among all samples, respectively.

Subsequently, by linearly fitting the relationship between normalized feature values and SHAP values (Figure S6), the influence coefficients $k_{ni}$ for key feature quantities were obtained. The slope of the fitted line represents the influence coefficient, whose physical significance is: positive values indicate positive correlation between the feature quantity and property, where increasing the feature quantity enhances target performance; negative values indicate negative correlation, where increasing the feature quantity reduces target performance; the absolute value reflects the strength of influence, with larger values indicating more significant impact on performance.

**S2.2 Element ranking criteria based on correlation**

Based on the positive or negative nature of influence coefficients $k_{ni}$, reasonable element selection criteria can be determined. For *K*_Q_ performance optimization, when key alloy factors are positively correlated with performance (such as Ω and Λ), elements with higher values in these features should be selected; conversely, for negatively correlated factors (such as PT4m, SL1m and JN1), elements with lower feature values should be selected.

**S2.3 Calculation of element ranking weight coefficients**

Considering the significant differences in the degree of influence of different key feature quantities on target properties, reasonable weight coefficients need to be assigned for element ranking of each feature quantity. The design of weight coefficients $Z_{ni}$ primarily considers two factors: First, the larger the SHAP value range of a key alloy factor $x_{ni}$, the greater its influence on target properties, and correspondingly, the weight coefficient for its element ranking should increase; Second, the stronger the monotonic relationship between the feature and target property, the higher the reliability of element ranking, and the weight coefficient should also be larger.

Since the influence coefficient $k_{ni}$ simultaneously reflects both factors, the normalized absolute value of influence coefficients was adopted as weights:

|  | $Z_{ni}=\frac{\vert k_{ni}\vert}{\sum_{i=1}^{m} \vert k_{ni}\vert}$ | (S4) |
| --- | --- | --- |

where m is the number of key alloy factors affecting a target property (5 feature for PCC-GB-K5).

**S2.4 Element importance ranking**

To achieve synergistic optimization of *K*_Q_, a weighted comprehensive method was adopted for overall element ranking. For each element, based on its order number $q_{i}$（$q_{i}$=1,2,3...） in each key feature quantity ranking and corresponding weight coefficient $Z_{ni}$, the weighted score was calculated:

|  | $S_{K_{Q}}=\sum_{i=1}^{5} Z_{ni}^{K_{Q}}\times q_{i}^{K_{Q}}$ | (S5) |
| --- | --- | --- |

where $S_{K_{Q}}$ represents the element's scores for fracture toughness performance. Lower scores indicate greater contribution to comprehensive performance. Based on this scoring system, the obtained element ranking results show that alloying elements ranked by their influence on enhancing *K*_Q_ properties in descending order are: Si, Nb, Ti, Hf, Zr, V, etc.

**S2.5 Selection and validation of candidate elements**

Based on the element ranking results and cost considerations, we selected Si, Nb, Ti, Zr, and V as the candidate elements. This data-driven element selection method demonstrates stronger scientific rigor and reliability compared to traditional empirical approaches. Through this systematic SHAP analysis method, we not only identified key factors affecting Nb-Si alloy properties but also established quantitative relationships from microscopic features to macroscopic properties, providing a scientific basis for experimental validation.

**Section S3. Comparison with the constrained mixture model**

The constrained mixture model was selected for comparison because it accounts for how the interconnected brittle silicide network constrains the deformation capacity of the ductile Nb solid solution (Nbss) matrix, which is the most relevant physical mechanism governing toughness in Nb-Si alloys.

The five validation alloys show varying microstructures, with the brittle silicide volume fraction (Vb) decreasing from alloy #1 to alloy #5, as shown in Figure S8. Vb values are 42.33%, 33.11%, 29.10%, 22.96%, and 10.56% for alloys #1 through #5, while the Nbss fractions are 57.67%, 66.89%, 70.90%, 77.04%, and 89.44%, respectively. These phase fraction data serve as the input parameter Vb in the constrained mixture model calculation.

The constrained mixture model captures the geometric constraint that the brittle silicide network places on the ductile Nbss phase. The governing equations are:

$\mathrm{Pc}(\mathrm{Vb})=\exp\left\{ -\frac{8q^{'}}{3}\left[ \frac{\mathrm{Vb}}{1-\mathrm{Vb}} \right] \right\}$ (S6)

$\text{K}_{\text{IC}}=\mathrm{Kb}\left[ 1+\text{α}\sqrt{1-\mathrm{Vb}}\left[ \left[ \frac{\mathrm{Kd}}{\mathrm{Kb}} \right]^{2}\mathrm{Pc}(\mathrm{Vb})-1 \right] \right]^{1/2}$ (S7)

where Vb is the brittle silicide volume fraction, Pc is the constraint factor that the brittle network imposes on the ductile phase, q' is an empirical parameter controlling constraint intensity (set to 1.0), α is a geometric correction factor (set to 0.5), Kb is the intrinsic toughness of the brittle phase (3.0 MPa·m^1/2^), and Kd is the intrinsic toughness of the ductile phase (32.0 MPa·m^1/2^).

The calculation results for all five alloys are given in Table S10. As Vb decreases from alloy #1 to #5, the constraint factor Pc increases from 0.1414 to 0.7299, meaning the brittle network exerts less constraint on the ductile matrix. The predicted *K*_IC_ values range from 7.781 to 18.929 MPa·m^1/2^, and the prediction error decreases from 40.89% for alloy #1 to 16.94% for alloy #5, with a mean error of 27.13% across the five alloys. The model performs better when the Nbss fraction is high because the geometric constraint assumption is better satisfied under ductile-dominant conditions. The comparison between the constrained mixture model and ML model is shown in Figure S9. The constrained mixture model produces predictions that follow the correct trend but with substantial underestimation for all five alloys, and the error is largest for alloys with high silicide fractions (#1 and #2) where microstructural interactions beyond simple geometric constraints are more significant. The ML model achieves a mean error of 3.1% across the five alloys, compared to 27.13% for the constrained mixture model. The gap in performance reflects the ML model's ability to capture nonlinear interactions among composition, phase fraction, and local microstructural features, which the single-mechanism constrained mixture model cannot represent.

**Section S4 Strengthening mechanisms**

Addition of alloying elements (Ti, V, Zr, Si) to Nb-based alloy systems produces different degrees of lattice distortion, mainly originating from atomic radius differences and elastic modulus mismatches between solute and matrix atoms. Systematic composition changes from samples #1 to #5 lead to formation of different volume fraction second phases, particularly achieving transformation from brittle Nb_3_Si and Nb_5_Si_3_ intermetallic phases toward more ductile Nbss phase. This phase evolution process significantly improves material mechanical properties. Main strengthening mechanisms of these optimized Nb-based alloys include solid solution strengthening and second phase strengthening. Compressive yield strength of optimized Nb-Ti-V-Zr-Si alloy systems can be determined through sum of contributions from independent strengthening mechanisms:

$\text{σ}_{\text{YS}}^{\text{cal}}\text{=}\text{σ}_{\text{0}}\text{+}\text{Δ}\text{σ}_{\text{ss}}\text{+}\text{Δ}\text{σ}_{\text{p}}$ (S8)

**S4.1 Solid solution strengthening**

Solid solution strengthening($\text{Δ}\text{σ}_{\text{ss}}$) serves as the primary reinforcement mechanism in most multi-element alloys. To quantify this effect, the $\text{Δ}\text{σ}_{\text{ss}}$ can be calculated using the subsequent formula^[32]^:

$\text{Δ}\text{σ}_{\text{ss}}\text{=}\left( \sum\left( \text{∆}\text{σ}_{\text{i}} \right)^{\text{3/2}} \right)^{\text{2/3}}$ (S9)

This strengthening primarily results from substantial atomic size differences and elastic modulus disparities. The strengthening values $\text{∆σ}_{\text{i}}$ derived from $\text{Δ}\text{σ}_{\text{ss}}$ can be categorized as:

$\text{∆σ}_{\text{i}}\text{=AG}{\text{f}_{\text{i}}}^{\text{4/3}}{\text{c}_{\text{i}}}^{\text{2/3}}$ (S10)

where *A* is an empirical constant of 0.02^[32]^. *G* denotes the shear modulus of these alloys. The mismatch parameter $\text{f}_{\text{i}}$ correlates with $\text{r}$ and *G*. They can be expressed as^[33]^:

$\text{f}_{\text{i}}\text{=}\sqrt{\text{δ}_{\text{G,}\text{ }\text{i}}^{\text{2}}\text{+}\text{α}^{\text{2}}\text{δ}_{\text{r,}\text{ }\text{i}}^{\text{2}}}$ (S11)

where $\text{δ}_{\text{r,}\text{ }\text{i}}$ denotes the atomic size mismatch and $\text{δ}_{\text{G,}\text{ }\text{i}}$ represents the shear mismatch. Both $\text{δ}_{\text{G,}\text{ }\text{i}}$ and $\text{δ}_{\text{r,}\text{ }\text{i}}$ can be expressed as:

$\text{δ}_{\text{G,}\text{ }\text{i}}\text{=}\frac{\text{9}}{\text{8}}\sum\text{c}_{\text{j}}\text{δ}_{\text{G,ij}}$ (S12)

$\text{δ}_{\text{r,}\text{ }\text{i}}\text{=}\frac{\text{9}}{\text{8}}\sum\text{c}_{\text{j}}\text{δ}_{\text{r,ij}}$ (S13)

Where $\text{c}_{\text{j}}$ represents the compositional fraction of element $\text{j}$. The $\text{δ}_{\text{G,ij}}$ and $\text{δ}_{\text{r,ij}}$ describe the modulus and size mismatches between elements $\text{i}$ and $\text{j}$. There are given by:

$\text{δ}_{\text{G,ij}}\text{=}\frac{\text{2}\left( \text{G}_{\text{i}}\text{-}\text{G}_{\text{j}} \right)}{\left( \text{G}_{\text{i}}\text{+}\text{G}_{\text{j}} \right)}$ (S14)

$\text{δ}_{\text{r,ij}}\text{=}\frac{\text{2}\left( \text{r}_{\text{i}}\text{-}\text{r}_{\text{j}} \right)}{\left( \text{r}_{\text{i}}\text{+}\text{r}_{\text{j}} \right)}$ (S15)

where $\text{G}_{\text{i}}$, $\text{G}_{\text{j}}$ are the shear moduli and $\text{r}_{\text{i}}$, $\text{r}_{\text{j}}$ are the atomic radii for elements $\text{i}$ and $\text{j}$, respectively.

Figure S12 shows the radar chart analyses of four key solid solution strengthening parameters for the five alloy samples. $\text{δ}_{\text{ri}}$ parameter shows atomic radius mismatch effects have small differences between samples, with values mainly distributed in -0.3 to 0.0 range, where Zr element exhibits relatively large negative value contributions; $\text{δ}_{\text{Gi}}$ parameter reflecting shear modulus difference effects shows obvious differences between samples, particularly significant numerical differences on Si and Zr element axes; $\text{f}_{\text{i}}$ parameter shows volume fraction contributions to solid solution strengthening are relatively uniform with small differences between samples; $\text{∆σ}_{\text{i}}$ parameter as final result of solid solution strengthening calculations demonstrates quantitative contributions of various elements to overall strengthening increments, with numerical ranges between 200 to 800, where sample #5 shows relatively high strengthening increment values on multiple element axes.

**S4.2 The** **second phase strengthening**

The Nb_3_Si and Nb_5_Si_3_ phases produced in these alloys increase their compressive yield strength. Such precipitation strengthening occurs via dislocation interactions following the Orowan mechanism. This enhancement can be quantified by determining the compressive yield strength increment caused by dislocation bypass around precipitate particles^[34]^:

$\text{∆}\text{σ}_{\text{p}}\text{=0.4M∙}\frac{\text{Gb}}{\text{πλ}\sqrt{\text{1-}\text{υ}}}\text{∙}\ln\left( \frac{\text{2}\text{r}\sqrt{\text{2/3}}}{\text{b}} \right)$ (S16)

Here, M=2.9 denotes the Taylor factor for BCC structures^[35]^. The geometric parameters are: *r* (mean precipitate dimensions), *λ* (inter-precipitate spacing), and *b* (Burgers vector magnitude). Material constants include *G* (shear modulus) and *υ* (Poisson's ratio). $\text{λ}$ and *b* are defined by^[36]^:

$\text{λ}\text{=}\left( \sqrt{\frac{\text{3}\text{π}}{\text{4}\text{f}}}\text{-1.64} \right)\text{r}$ (S17)

$\text{b=}\sqrt{\text{3}}\text{α}\text{/2}$ (S18)

where $\alpha$ denotes the lattice parameter, *f* is the Laves phase proportion.

Figure S13 presents quantitative decomposition and contribution analysis of strengthening mechanisms for five validation alloy samples. Figure S13a indicates good overall agreement between experimental and theoretically calculated values, consistent with the strengthening models. Combined with the strengthening mechanism proportion analysis Figure S13c, solid solution strengthening ($\text{Δ}\text{σ}_{\text{ss}}$) dominates in all samples, with proportions gradually decreasing from 84% in sample #1 to 68% in sample #5; basic strength ($\text{σ}_{\text{0}}$) proportions increase from 15% to 30%; precipitation strengthening ($\text{Δ}\text{σ}_{\text{p}}$) contributions remain at low levels (1-2%).

The intrinsic yield strength $\text{σ}_{\text{0}}$ element contribution analysis in Figure S8b shows that Nb element contributions increase from 78MPa to 92.4MPa, Ti element from 63.38MPa to 75.08MPa, Si element maintains stable 50.4MPa levels, Zr element increases from 66.3MPa to 72.75MPa, and V element varies from 81.6MPa to 88.8MPa. This systematic analysis of strengthening mechanisms reveals evolution patterns of various strengthening mechanisms for the alloys, and provides a basis for controlling the material strengthening mechanisms to guide subsequent design.

**Section S5. Processing parameter analysis and model applicability**

**S5.1 Processing parameter statistics**

The dataset is restricted to arc-melted samples drawn from 216 literature sources. To characterize the processing conditions represented in the training data, nine key parameters were extracted: remelting cycles, ingot mass, ingot diameter, ingot thickness, chamber argon pressure, protective atmosphere, cooling method, raw material purity, and melting current. The complete extraction database is given in Table S11, and the summary statistics are presented in Table S12.

Remelting cycles were documented for 206 samples (95.4% coverage), ranging from 3 to 8 cycles with mean 5.0 ± 0.8 and median of 5. Most samples (53.9%) used exactly 5 remelts, while 96% exceeded 4 remelts, a threshold above which compositional homogeneity in niobium-silicide systems is generally considered sufficient. Ingot mass was documented for 182 samples (84.3% coverage), with nominal range 65–2500 g but clustering around 100–150 g (63.7% of values), with mean 198 ± 352 g and median 110 g. Ingot geometry showed 70.4% documentation for both diameter and thickness (152 samples each), with 86.2% measuring 33–35 mm diameter by 89.5% at 8–10 mm thickness. All samples used argon as the protective atmosphere (198 samples explicitly stated, 98% documentation), and chamber argon pressure was recorded for 152 samples (70.4% coverage), ranging nominally from 0.03 to 0.8 MPa, with 84.2% using 0.05 MPa and another 11.2% using 0.03 MPa, yielding median 0.05 MPa. All 203 samples used water-cooled copper crucibles. Raw material purity was documented for 93 samples (43.1% coverage), predominantly 99.95% (74.2% of documented values) with median 99.95%. Melting current was documented for 82 samples (38.0% coverage), mostly 300–600 A (98.8% of documented values).

**S5.2 Effect of processing variability on *K*_Q_**

Pearson correlation analysis between each documented processing parameter and fracture toughness reveals no statistically significant relationships except for raw material purity (r = −0.287, p = 0.005) and ingot mass (r = 0.194, p = 0.009), as shown in Figure S17. The remaining parameters including remelting cycles, ingot diameter, ingot thickness, argon pressure, and melting current all showed p > 0.05.

The total *K*_Q_ variance is *σ*_total_ = 4.02 MPa·m^1/2^. The PCC-GB-K5 model achieves training R² = 0.98 and test R² = 0.93, with training MAE = 0.77 MPa·m^1/2^ and test MAE = 2.06 MPa·m^1/2^. Using test R² = 0.93, the explained variance is $\text{σ}_{\text{comp}}\text{=}\sqrt{\text{R}^{\text{2}}\text{×}\text{σ}_{\text{total}}^{\text{2}}}\text{=3}\text{.98}\text{MPa·}\text{m}^{\text{1/2}}$, and the unexplained variance is $\text{σ}_{\text{unexplained}}\text{=}\sqrt{\text{(1-}\text{R}^{\text{2}}\text{)×}\text{σ}_{\text{total}}^{\text{2}}}\text{=1.06MPa·}\text{m}^{\text{1/2}}$, which encompasses both processing variations and measurement uncertainties. Within-group analysis confirms limited processing contribution. Among samples with ≥4 remelting cycles (n=198, 96.1% of dataset), *K*_Q_ standard deviation is 3.05 MPa·m1/2 compared to 3.09 MPa·m1/2 for the full dataset, indicating remelting variations contribute <4%. Standard small ingots (n=131, 86.2% of diameter-documented samples) show similarly negligible *K*_Q_ standard deviation differences.

Based on these results, we estimate processing variations contribute approximately 0.4-0.5 MPa·m^1/2^ (~1-2% of total variance), with measurement uncertainties accounting for ~0.2 MPa·m^1/2^ (~0.2%), yielding combined processing and measurement contribution of 0.45-0.54 MPa·m^1/2^ (approximately 1-2% of total variance). The variance decomposition is summarized in Table S13.

**S5.3 Model applicability**

The training dataset exhibits relatively consistent processing conditions, with 96% of samples receiving ≥4 remelting cycles (median 5), 86% using small ingot geometry (33–35 mm diameter, 8–10 mm thickness) yielding cooling rates of 1.2–1.6 K/s, 84% employing 0.05 MPa argon pressure, all samples using argon atmosphere and water-cooled copper crucible solidification, and documented samples showing predominantly 99.95% raw material purity. These conditions correspond to established compositional homogeneity (>95% from 4+ remelts) and controlled oxygen contamination (estimated 50–200 ppm).

Model predictions are reliable when applied to alloys processed under conditions consistent with this training dataset. Applicability becomes uncertain under deviations: samples with <4 remelting cycles may exhibit compositional inhomogeneity (>5% local variation) not captured by the composition-based model; cooling rates substantially faster (>3 K/s) or slower (<0.3 K/s) may alter grain size, silicide morphology, and phase distribution in ways not reflected in bulk composition; oxygen contamination exceeding 300 ppm can introduce brittle oxide phases and reduce toughness beyond training experience; and alternative processing routes including powder metallurgy, directional solidification, or additive manufacturing produce fundamentally different microstructures not represented in the arc-melted training dataset.

For experimental validation, we recommend performing ≥4 remelting cycles (preferably 5–6), using water-cooled copper crucible arc-melting with argon atmosphere at 0.04–0.06 MPa, employing ingot dimensions of 30–40 mm diameter and 8–12 mm thickness to achieve 1–2 K/s cooling rates, using raw materials with minimum 99.5% purity, and maintaining oxygen contamination below 200 ppm through proper chamber preparation and high-purity argon (≥99.999%). Under these conditions, model predictions exhibit uncertainty of approximately ±1.0 MPa·m^1/2^ from natural compositional scatter and residual processing variations, representing ~25% of the σunexplained upper bound (1.06 MPa·m^1/2^) and confirming that compositional factors dominate (~98%) the total *K*_Q_ variance.

**References**

[1] Z. Guo, X. Shen, F. Liu, J. Guan, Y. Zhang, F. Dong, Y. Wang, X. Yuan, B. Wang, L. Luo, Y. Su, J. Cheng, *J. Alloys Compd.* **2023**, *960*, 170739.

[2] C. M. Lin, C. C. Juan, C.H. Chang, C.W. Tsai, J. W. Yeh, *J. Alloys Compd.* **2015**, *624*, 100.

[3] B. Yu, J. Chen, N. Ding, X. Yang, W. Bao, Z. Cai, G. Xie, *Mater. Sci. Eng., A* **2024**, *915*, 147223.

[4] Z. Li, L. Wang, B. B. Wang, S. S. Li, Z. W. Li, L. S. Luo, R. R. Chen, Y. Q. Su, J. J. Guo, *Mater. Sci. Eng., A* **2022**, *844*, 143072.

[5] S. Ge, H. Fu, L. Zhang, H. Mao, H. Li, A. Wang, W. Li, H. Zhang, *Mater. Sci. Eng., A* **2020**, *784*, 139275.

[6] Y. Liu, Y. Zhang, H. Zhang, N. Wang, X. Chen, H. Zhang, Y. Li, *J. Alloys Compd.* **2017**, *694*, 869.

[7] N. Y. Yurchenko, N. D. Stepanov, D. G. Shaysultanov, M. A. Tikhonovsky, G. A. Salishchev, *Mater. Charact.* **2016**, *121*, 125.

[8] Y. C. Liao, W. T. Ye, P. S. Chen, P. H. Tsai, J. S. C. Jang, K. C. Hsieh, C. Y. Chen, J. C. Huang, H. J. Wu, Y. C. Lo, C. W. Huang, I. Y. Tsao, *Intermetallics* **2021**, *135*, 107213.

[9] H. Zhang, Y. Du, L. Lai, N. Guo, N. Li, S. Guo, *J. Alloys Compd.* **2023**, *932*, 167675.

[10] Z. Q. Xu, Z. L. Ma, M. Wang, Y. W. Chen, Y. D. Tan, X. W. Cheng, *Mater. Sci. Eng., A* **2019**, *755*, 318.

[11] Z. Q. Xu, Z. L. Ma, Y. Tan, M. Wang, Y. Zhao, X. W. Cheng, *J. Alloys Compd.* **2022**, *900*, 163517.

[12] X. Li, H. Li, Q. Li, C. Jin, K. Hua, H. Wang, *Mater. Charact.* **2022**, *189*, 111921.

[13] K. Gao, J. Z. Wang, Y. H. Meng, Y. Li, Y. Zhang, *Intermetallics* **2022**, *148*, 107622.

[14] C. Li, S. H. Chen, Z. W. Wu, Z. F. Zhang, Y. C. Wu, *J. Mater. Res.* **2022**, *37*, 1664.

[15] L. Xiang, W. Guo, B. Liu, A. Fu, J. Li, Q. Fang, Y. Liu, Microstructure and Mechanical Properties of TaNbVTiAlx Refractory High-Entropy Alloys. In *Entropy*, **2020**; Vol. 22.

[16] N. D. Stepanov, N. Y. Yurchenko, D. G. Shaysultanov, G. A. Salishchev, M. A. Tikhonovsky, *Mater. Sci. Technol.* **2015**, *31*, 1184.

[17] M. Wang, Z. L. Ma, Z. Q. Xu, X. W. Cheng, *Mater. Sci. Eng., A* **2021**, *808*, 140848.

[18] K. Lee, Y. Jung, J. Han, S. H. Hong, K. B. Kim, P. K. Liaw, C. Lee, G. Song, Development of Precipitation-Strengthened Al0.8NbTiVM (M = Co, Ni) Light-Weight Refractory High-Entropy Alloys. In *Materials*, **2021**; Vol. 14.

[19] V. Bhardwaj, Q. Zhou, F. Zhang, W. Han, Y. Du, K. Hua, H. Wang, *Tribol. Int.* **2021**, *160*, 107031.

[20] Z. D. Han, H. W. Luan, X. Liu, N. Chen, X. Y. Li, Y. Shao, K. F. Yao, *Mater. Sci. Eng., A* **2018**, *712*, 380.

[21] C. Zhu, X. Li, Z. Zhang, *Vacuum* **2024**, *221*, 112888.

[22] S. Y. Chen, X. Yang, K. A. Dahmen, P. K. Liaw, Y. Zhang, Microstructures and Crackling Noise of AlxNbTiMoV High Entropy Alloys. In *Entropy*, **2014**; Vol. 16, pp 870.

[23] S. Lin, W. Lai, F. Vogel, X. Tong, D. You, W. Li, X. Wang, *Int. J. Refract. Met. Hard Mater* **2023**, *116*, 106361.

[24] W. Yang, S. J. Pang, G. Wang, Y. Liu, P. K. Liaw, T. Zhang, *Rare Met.* **2022**, *41*, 2305.

[25] W. Wang, Z. Zhang, J. Niu, H. Wu, S. Zhai, Y. Wang, *Mater. Today Commun.* **2018**, *16*, 242.

[26] N. D. Stepanov, N. Y. Yurchenko, D. V. Skibin, M. A. Tikhonovsky, G. A. Salishchev, *J. Alloys Compd.* **2015**, *652*, 266.

[27] S. Tao, W. Jiang, W. Zhang, H. Qiu, S. Wu, S. Guo, B. Zhu, *J. Alloys Compd.* **2022**, *928*, 166986.

[28] Y. Huang, X. Yu, L. Deng, Y. Gao, S. Wang, B. Wang, *J. Mater. Res. Technol.* **2023**, *23*, 2824.

[29] N. Yurchenko, V. Mirontsov, E. Mishunina, N. Stepanov, *Mater. Charact.* **2025**, *222*, 114856.

[30] D. Qiao, H. Liang, S. Wu, J. He, Z. Cao, Y. Lu, T. Li, *Mater. Charact.* **2021**, *178*, 111287.

[31] N. N. Guo, L. Wang, L. S. Luo, X. Z. Li, R. R. Chen, Y. Q. Su, J. J. Guo, H. Z. Fu, *J. Alloys Compd.* **2016**, *660*, 197.

[32] O. N. Senkov, J. M. Scott, S. V. Senkova, D. B. Miracle, C. F. Woodward, *J. Alloys Compd.* **2011**, *509*, 6043.

[33] W. Huang, J. Hou, X. Wang, J. Qiao, Y. Wu, *Intermetallics* **2022**, *151*, 107735.

[34] F. Haftlang, H. S. Kim, *Mater. Des.* **2021**, *211*, 110161.

[35] U. F. Kocks, *Metallurgical Transactions* **1970**, *1*, 1121.

[36] P. Wang, H. Cai, S. Zhou, L. Xu, *J. Alloys Compd.* **2017**, *695*, 462.
